# People prefer to negotiate with women, even when outcomes are identical and gender is unknown

**DOI:** 10.1073/pnas.2523202123

**Published:** 2026-06-22

**Authors:** Charlotte H. Townsend, Laura J. Kray, Solène Delecourt

**Affiliations:** ^a^ Haas School of Business, University of California Berkeley, Berkeley, CA 94720; ^b^ Industrial & Labor Relations School, Cornell University, Ithaca, NY 14853

**Keywords:** gender, negotiations, gender differences

## Abstract

While gender and economic outcomes in negotiation have been studied for decades, much less is known about gender differences in subjective outcomes, such as trust, rapport, and willingness to negotiate again, despite their importance for long-term success. In a series of studies with over 2,000 participants, we find that people consistently report better subjective outcomes when negotiating with women. This preference persists even in anonymous negotiations where gender is unknown and cannot be inferred from behavior, as well as in conditions where negotiation partner gender is randomly assigned. Importantly, we find no gender difference in economic outcomes. To understand why, we analyzed transcripts of real negotiations for any behavioral differences. These findings reveal an overlooked gender dynamic: While men and women achieve similar economic results, women foster stronger interpersonal relationships, which in turn lead to greater satisfaction and greater desire for future negotiations with women.

## Significance

This research advances our understanding of negotiations by highlighting women’s advantages in the social consequences of negotiations. Negotiations span a wide range of contexts: from formal job offers to the daily division of household tasks, each with consequences beyond the immediate exchange. We find that women are liked more than men by their negotiation partners, which in turn increases partner satisfaction and heightens desire for future negotiations with women, even when anonymous. Importantly, women achieve economic outcomes on par with men, suggesting that greater likability does not come at a performance cost and may provide women with more opportunities to negotiate that compound into economic gains. These findings offer a counterpoint to narratives that emphasize only women’s disadvantages in negotiations.

Negotiations are discussions between two or more parties with the goal of reaching an agreement, in which parties may have a variety of aligned or conflicting interests (1). Negotiations broadly encompass job salaries, descriptions and roles, resource allocations, and conflict resolution, and include more day-to-day negotiations such as conversations to determine the price of a good or service, or to redistribute the household division of labor (2, 3). Because negotiations span such a variety of meaningful conversations, the implications of successful negotiation skills are far-reaching (4).

Prior work has suggested that gender differences in outcomes may arise because women are less likely to initiate negotiations and achieve worse economic outcomes (5–9). However, gender effects in negotiations have been inconsistent with early theorizing, which suggested that men are consistently better negotiators than women (10). Negotiation researchers have since discovered several moderators of gender effects (3, 11, 12), with each subsequent review categorizing and enumerating new contextual moderators of gender differences in negotiation outcomes. While gender effects on economic outcomes in negotiations have been extensively examined, the role of gender in subjective value has been largely overlooked (13).

Subjective value refers to the social and emotional consequences of negotiations and can sometimes prove more important than economic value (14–16). Subjective value predicts the desire to negotiate again with the same counterpart (17), which is essential for building lasting relationships. Furthermore, subjective value predicts long-term satisfaction more strongly than economic outcomes (16, 18). Subjective value and economic outcomes are also not mutually exclusive (19). Prior research demonstrates that subjective value in the first round of a multiround negotiation correlates with economic performance in the second round, even when controlling for first-round economic outcomes. This suggests that creating subjective value in early negotiations can yield long-term financial rewards. To this end, prior researchers have examined the desire to negotiate again with the same counterpart as a consequential outcome of negotiations (15, 20), aligning with the logic that repeat customers and businesses are associated with higher revenue (21). They argue that the economic value derived from subjective value is realized in the long-term, as subjective value creates more economic value in subsequent negotiations and more opportunities for future negotiations (15). Further, economists have demonstrated the long-term economic advantage of cooperation in prisoner’s dilemma games (22–24). In such games, a cooperative strategy surpasses others over time even though it does not outperform alternative strategies in any single interaction. As argued by Nobel laureate Richard Thaler, cooperative behavior can enhance economic outcomes because “a cooperative act itself may with high probability be reciprocated with cooperation, to the ultimate benefit of the cooperator” (25). Relatedly, negotiators with positive reputations also achieve better economic outcomes (26, 27). Recent work by Hart and Schweitzer has further illuminated how subjective value positively affects economic outcomes and postnegotiation behavior (28, 29). Given these important implications of subjective value, understanding whether gender differences exist, and in what direction, is crucial. Further, recent research has called for examining subjective outcomes to better understand women’s experiences in negotiations (30, 31).

Subjective value in negotiations stems from the social perception literature (15, 32), which demonstrates that perceptions form from both observed stimuli and our biases (33, 34). In other words, perceptions are based on actual behavior but can also be influenced by stereotypes. In negotiation research, gender stereotypes have been shown to impact perceptions of competence, who can initiate negotiations, and who can display certain emotions (5, 35, 36). Real gender differences in negotiation behavior also exist, including differences in ethical behavior (37, 38), avoiding impasses (39), and propensity to negotiate (31, 40). Both gender stereotypes and actual behavioral differences may contribute to a gender difference in subjective value.

## The Case for Women’s Advantage in Subjective Value.

Women may possess an advantage in subjective value, due to gender stereotypes, and/or behavioral differences. Gender stereotypes about women may lead them to receive higher subjective value ratings in negotiations, given the content of those stereotypes. Women are expected to be warmer and more communal than men (41, 42). Perceptions of warmth and communality are especially relevant to social perceptions of others (43, 44). People place greater weight on the communal dimension of stereotypes when forming perceptions of others (45). Furthermore, women are perceived as higher in friendliness, trustworthiness, and likeability across contexts (46). Consistent with this reasoning, previous work has coined the “women are wonderful” effect, in which women are evaluated more favorably than men (47). Therefore, these social perceptions may contribute to higher subjective value in negotiations with women.

Aside from stereotypes, prior work has shown that women are more relationally oriented (48, 49) and are therefore expected to create more positive impressions in negotiations (50). While both men and women equally value the economic aspect of negotiations, women value the relational aspect of negotiations more than men (51–54) and are consequently more likely to reach a deal when they have weak alternatives (39). Kray and Gelfand (2009) also found that women experienced more relief after their first offer was accepted, possibly because they valued their relationship with their partner more than men did. In line with this relational emphasis, initial evidence shows that women tend to be rated higher on important components of subjective value, such as trustworthiness (55, 56), and place greater value on subjective outcomes (57). Women also tend to be more cooperative (9) and are more likely to develop reputations as cooperative negotiators compared to men (58). Negotiators with positive reputations for cooperation are able to leverage their expertise successfully (26). Women also consistently demonstrate greater concern for ethics and maintain higher ethical standards (59, 60), which is important for negotiation satisfaction (15). When negotiators use deception, they undermine subjective value in both current and subsequent negotiations (17). Based on this reasoning, we anticipate that women negotiation partners will be associated with greater subjective value in negotiations.

Across five studies (*N* = 2,401), we examine gender and subjective value in negotiations, and the downstream consequences of the desire for future negotiations and satisfaction. In Study 1, we test support for the hypothesized women’s cumulative advantage in subjective value over a 10-wk negotiation course. In Studies 2 through 4, we disentangle differences in stereotypes and behaviors to investigate the root causes of these partner-based gender effects and their generalizability. We examine whether a gender gap exists in subjective value and, if so, whether it can be attributed to differences in behavior, stereotypes, or both. In Study 5, we analyze negotiation transcripts to identify behavioral gender differences that may contribute to the gender gap in subjective value.

## Results

### Study 1.

We used an archival dataset of existing measures from a full-time MBA negotiation course. The dataset includes over 2,000 observations from 231 students. In this course, students completed face-to-face negotiation role-playing exercises followed by postnegotiation online surveys every week. Students could participate in up to 10 negotiations, and on average, students provided feedback on 9.82 occasions and received feedback 9.57 times. Students were randomly assigned their negotiation roles and negotiation partners. Students debriefed the negotiations after they took place and before completing the survey. In the postnegotiation surveys, students evaluated their partners on subjective value measures (e.g., “How effective was your partner at building trust?” from 1 to 7), and the desire to negotiate again with the same partner [“Given the way your partner negotiated, would you want to work with your partner again?” Yes or no (18)]. We also had independent Research Assistants code the economic outcome value for each negotiation with economic outcomes, by distributive outcomes (e.g., salary, bonus, sale price, etc.) and the extent to which the outcome was integrative, or the extent to which the agreement included shared interests for mutual gains (0 if no agreement, 1 if nonintegrative, and 2 if integrative).

#### Subjective value.

*SI Appendix*, Tables S1 and S2 include the full results. We found several significant differences in partner evaluations, such that people rated women as higher in building trust (*b* = −0.17, *t*(2,231) = −3.52, *P* < 0.001), fairness (*b* = −0.21, *t*(2,250) = −4.42, *P* < 0.001), satisfying their partner’s needs (*b* = −0.22, *t*(2,252) = −3.74, *P* < 0.001), expanding the pie (*b* = −0.12, *t*(2,220) = −2.16, *P* = 0.031), communicating (*b* = −0.17, *t*(2,218) = −3.77, *P* < 0.001), listening (*b* = −0.21, *t*(2,237) = −4.71, *P* < 0.001), and overall (*b* = −0.14, *t*(2,224) = −3.25, *P* = 0.001), suggesting that women created more subjective value in negotiations. There was no partner gender difference in satisfying their own interests (*b* = 0.05, *t*(2,196) = 1.03, *P* = 0.305) or competitiveness (*b* = 0.11, *t*(2,163) = 1.91, *P* = 0.057).

#### Specification curve analysis.

The analyses with subjective value measures were not preregistered; therefore, our analytical choices may not be free from bias and could skew our results toward statistically significant effects. Thus, we also used specification curve analysis to address potential “researcher degrees of freedom” (61, 62). This analysis method enables us to test various model specifications consistent with our theoretical framework. Of the 36 effects, 36 are consistent across specifications, and 28 are significant (linear: *n* = 18, *Mdn B* = 0.174, SD = 0.107; multilevel: *n* = 18, *Mdn B* = 0.165, SD = 0.115). All of the nonsignificant effects are analyses with variables that we report as nonsignificant in the main results. These analyses demonstrate that the reported results are consistent across computations. *SI Appendix*, Figs. S1 *A* and *B* and S2 *A* and *B* display the effect estimates for each model specified.

#### Desire for future negotiations.

Participants were significantly more likely to want to negotiate with women partners again compared to men (*b* = −0.43, *P* = 0.019). Participants indicated they would like to negotiate with men, 91.9 percent, compared to 94.4 percent of women.

#### Mediation.

To explore whether the gender gap in subjective value contributed to the participants wanting to negotiate again with women more than men, we conducted a parallel mediation (63) of partner gender on the desire to negotiate with said partner again, with ratings of building trust, being fair, satisfying partner interests, expanding the pie, communication, listening, and overall effectiveness as mediators. We found that building trust, being fair, and satisfying partner interests contributed to the gender gap (building trust: *b*indirect = −0.003, 95% CI [−0.005, −0.001], *z* = −2.58, *P* = 0.010; being fair: *b*indirect = −0.005, 95% CI [−0.008, −0.002], *z* = −3.39, *P* < 0.001; satisfying partner interests: *b*indirect = −0.003, 95% CI [−0.005, −0.001], *z* = −2.54, *P* = 0.011), such that women were rated higher on these subjective value measures which contributed to a greater desire for future negotiations. Expanding the pie, communication, listening, and overall effectiveness ratings did not significantly mediate the effect (*Ps* > 0.09).

#### Economic outcomes.

We did not find a significant participant gender difference in distributive outcomes (*b* = 0.058, *t*(210) = 0.82, *P* = 0.415). We also did not find a significant partner gender difference in distributive outcomes (*b* = 0.002, *t*(1,024) = 0.03, *P* = 0.978). We also found no significant difference in integrative outcomes by participant gender (*X^2^* (2, *N* = 231) = 2.08, *P* = 0.353) or partner gender (*X^2^* (2, *N* = 231) = 1.71, *P* = 0.425).

### Study 2.

In Study 1, participants were aware of their partner’s gender, which poses a significant limitation. We do not know whether these effects are due to actual gender differences in behavior or merely reflect gender stereotypes. In Study 2, we sought to identify the source of the gender differences observed in Study 1. We used an archival dataset from previously published research, available at https://huggingface.co/datasets/casino (64). The authors recruited participants from Amazon Mechanical Turk, and the final sample consisted of 1,030 dyads with 846 unique participants. The sample had 472 women, 372 men, and two participants who did not indicate either gender. All the remaining demographic information is reported in the *Materials and Methods*. Participants completed a presurvey and then took part in an online negotiation, in which they were randomly paired and could communicate via chat to reach a deal. Although the negotiation involved fictitious campfire resources, participants were incentivized with a performance-based bonus of 8.33 cents per point, with a maximum of 36 points. Once they agreed on a deal or reached an impasse, they completed a postsurvey questionnaire. In the postnegotiation survey, participants completed a subjective value measure (“How much do you like your opponent?”) and indicated their satisfaction with the outcome (“How satisfied are you with the negotiation outcome?”). Participants’ economic outcomes were based on their final deal, and joint points were calculated as the sum of points earned by both partners.

### Pretest.

Before diving into the results of Study 2, we wanted to confirm that participants could not infer their counterparts’ gender. Previous research has found that individuals may be able to reasonably determine others’ gender online, even when it is not explicitly provided, by viewing their online seller profiles and making inferences based on language, product choices, and usernames (65, 66). Participants did not indicate what they believed to be the gender of their partner, which leaves open the possibility that individuals who took part in these negotiations inferred, correctly or incorrectly, their partner’s gender, which affected their evaluations of their negotiation partner due to gender stereotypes. To examine whether the negotiation chat conversations could be informative of the agents’ gender, we recreated and displayed the real chat negotiations from participants in Study 2 to a new sample. We recruited undergraduates at a large research university who completed the survey for course credit. Thus, the sample size was determined by the number of students enrolled in the course. All participants in the study completed the survey during the Spring Semester of 2023. Following the preregistration, we excluded 65 participants who indicated they did not read the survey carefully. The final sample consisted of 180 participants (97 women, 81 men, and two nonbinary individuals). All the remaining demographic information is reported in the *Materials and Methods*.

The procedure was based on prior work, in which participants tried to infer and indicate the gender of someone online when gender information was not explicitly provided (65, 66). We randomly selected a subset of 100 dyads and displayed their conversations to participants. All the agents in the subset of conversations self-identified as either a woman or a man. Each participant was shown five of the 100 possible conversations. For an example of a conversation, see *SI Appendix*, Fig. S3. Participants were asked, “Please evaluate which gender you believe each agent is,” and the response options were “Woman,” “Man,” or “Cannot discern.”

A binomial test indicated that the proportion of correct inferences of 0.43 was lower than chance (*P* < 0.001), suggesting people could not infer gender from the transcripts. Preregistered as an exploratory analysis, we also conducted analyses excluding Cannot discern responses from the data. We found that the proportion of correct inferences of 0.52 was not significantly different from chance (*P* = 0.109). In exploration, we examined whether there was an association between the inferred gender and accuracy. There was no significant relationship between the two variables (*X^2^* (1, *N* = 180) = 0.89, *P* = 0.347). People were not more accurate about inferring the gender of men or women agents. Table 1 includes counts of the gender that participants inferred or could not discern, and whether their inferences were correct or incorrect.

**Table 1.** Participants were unable to infer gender better than chance (Study 2 pretest)

	Correct inference	Incorrect inference
Inferred woman	390	376
Inferred man	391	342
Cannot discern	0	301

#### Subjective value.

Returning to the results of Study 2, we found that women were liked significantly more by their partners than men (*b* = −0.11, *t*(1,882) = −2.28, *P* = 0.023), even when controlling for points (*b* = −0.11, *t*(1,921) = −2.23, *P* = 0.026), and even when controlling for additional partner demographic information: age, ethnicity, and education.

#### Satisfaction.

There was no significant partner gender difference in reported satisfaction (*b* = −0.06, *t*(2,017) = −1.34, *P* = 0.181).

#### Mediation.

However, we found that women partners were liked more, and greater liking was associated with greater satisfaction (*b*indirect = −0.073, 95% CI [−0.135, −0.013], *z* = −2.33, *P* = 0.020).

#### Economic outcomes.

**Fig. 1.**
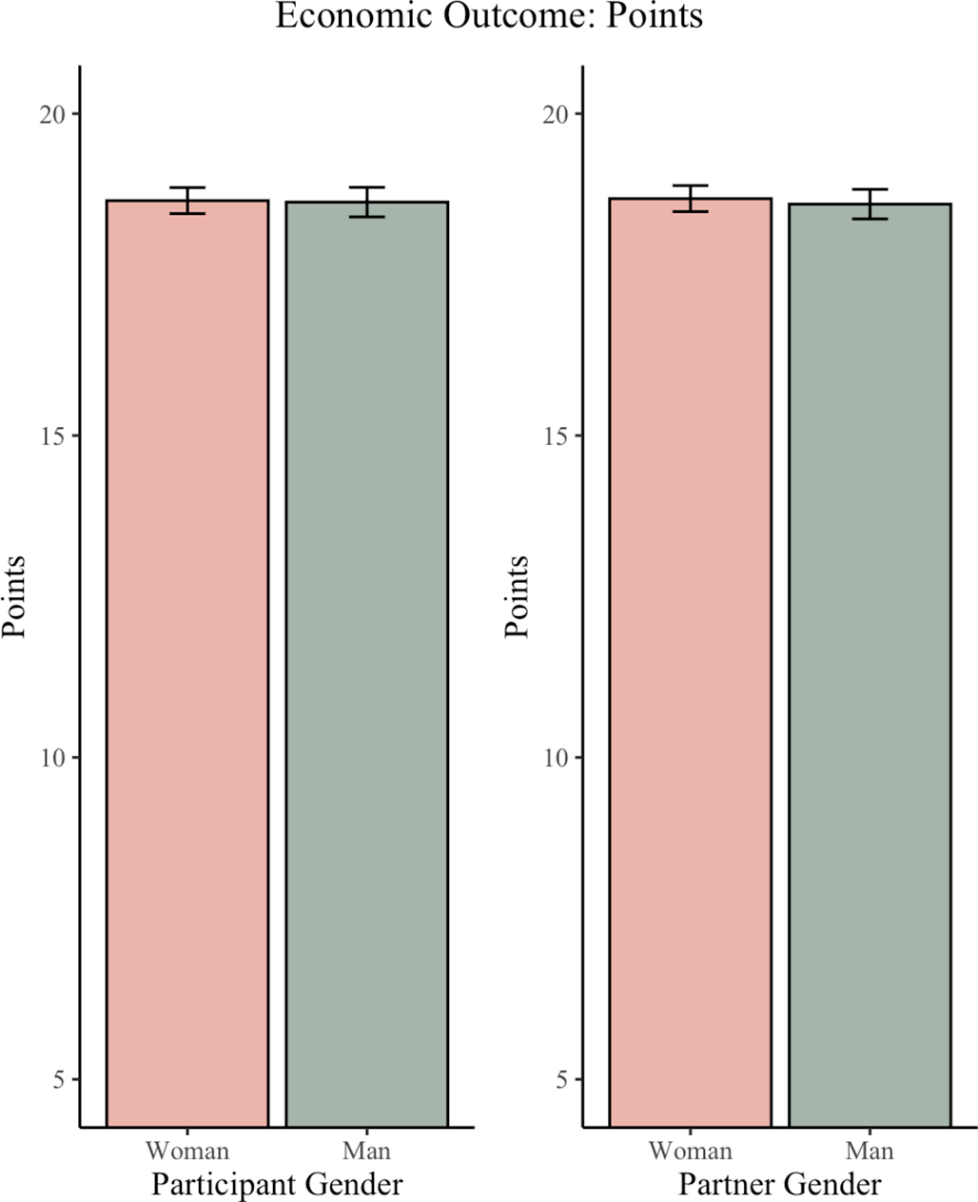
Women and men achieve the same economic outcomes (Study 2). Note. The error bars are 95% CI.

We did not find significant participant or partner gender differences in economic outcomes (participant gender: *b* = −0.02, *t*(2,053) = −0.13, *P* = 0.897; partner gender: *b* = −0.09, *t*(2,053) = −0.55, *P* = 0.586). We also did not find a significant participant or partner gender difference in joint points (participant gender: *b* = −0.11, *t*(2,053) = −0.50, *P* = 0.617; partner gender: *b* = −0.11, *t*(2,053) = −0.50, *P* = 0.617), such that there was no gender difference in value creation. Fig. 1 displays the economic outcomes, points, by participant and partner gender.

Therefore, the gender gap in subjective value remains even when partners are anonymous, and there is no gender gap in economic outcomes.

### Study 3.

We previously found evidence of a preference for women negotiators that persists even when negotiators remain anonymous to one another. This raises a critical question: when gender information is available, do the gender differences in subjective value stem from actual behavioral differences, or are they primarily driven by gender-based stereotypes? And if so, are these behavioral differences greater than perceptions in driving these results?

To investigate the role of stereotypes, we draw on the stereotype content model (42, 67), which identifies two dimensions underlying group stereotypes and social impressions: warmth and competence. According to this model, women are stereotypically perceived as higher in warmth but lower in competence relative to men. If these stereotypes influence subjective value, such that women negotiators are perceived as warmer but less competent than men, then this would provide compelling evidence that stereotypes, rather than behavioral differences alone, drive the preference for women negotiators.

We designed an experiment to disentangle the causal effects of negotiators’ actual gender from their perceived gender. Our study has three primary objectives. First, we attempt to replicate our previous online chat negotiation findings using third-party observers, testing whether agents who self-identify as women create greater subjective value, as in, they are liked more and generate greater partner satisfaction than self-identified men, even without explicit gender information. Second, we examine whether partner gender influences perceptions of warmth and competence, both measured using item composites, as well as willingness to negotiate with the same partner again. Finally, we investigate whether individuals’ actual gender predicts subjective value above and beyond the effect of perceived gender.

We recruited 800 U.S. participants from Prolific and 803 participants completed this experiment. Per our preregistration, we excluded 30 participants who self-reported that they did not read each conversation carefully. The final sample consisted of 773 participants (372 women, 383 men, 13 nonbinary individuals, and five who preferred not to answer). All the remaining demographic information is reported in the *Materials and Methods*.

**Fig. 2.**
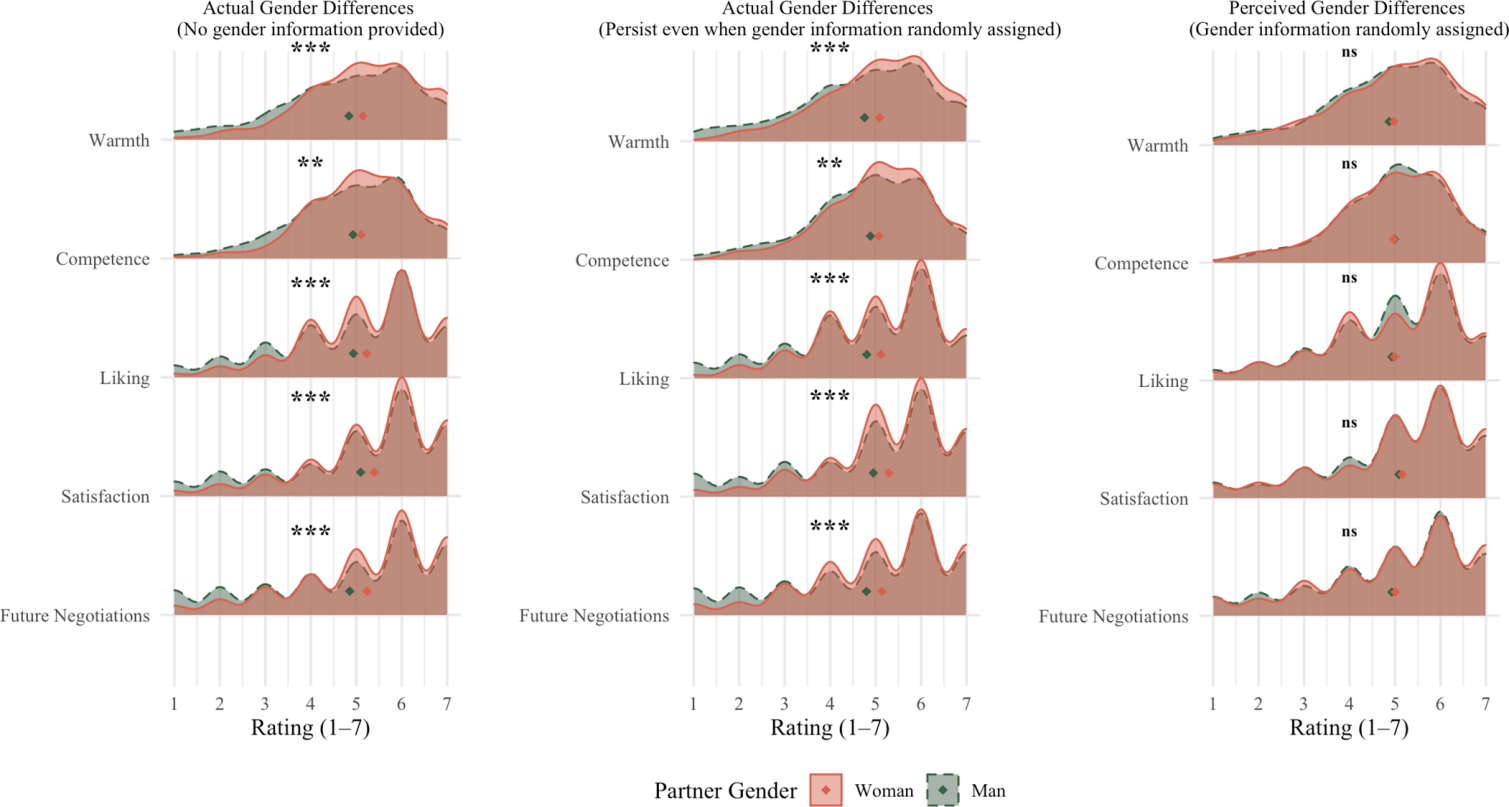
Women partners were evaluated significantly higher than men partners across all key measures, even when targets were randomly assigned gender (Study 3). Note. Distributions of key measures by partner gender. Actual gender is the self-reported gender of partners, and perceived gender is the partner’s gender that was randomly assigned and displayed to participants (i.e., “your partner is a woman”). The first panel displays actual gender differences in partner ratings, the second displays actual gender differences in partner ratings even when gender information is randomly assigned to participants, and the third displays perceived gender differences in partner ratings based on the partner gender that was randomly assigned to participants. Diamonds indicate mean ratings for each partner gender. Asterisks indicate significant differences between partner gender ratings ***P < 0.001, **P < 0.01.

Participants were given a link to complete the survey, in which they read recreated chat negotiations using the same dialogues from the online negotiation study (Study 2). The procedure was similar to the gender inference study (Study 2 pretest), such that each participant was shown a random subset of five of the 100 possible conversations, except that we manipulated the gender information provided. Participants were randomly assigned to imagine they were one of the agents in the conversation. The study was a one-factor (gender information) design with three levels (no label, man label, woman label). Participants either read conversations with no gender information or had the target in the conversations randomly assigned a gender (between subjects). After reading each conversation, participants provided their perceptions of their partner and the negotiation. Fig. 2 displays the distributions of key measures.

#### Control condition (test of actual gender differences).

##### Stereotype content.

Replicating and extending the results of Study 2, which were based on first-person negotiation experiences, to third-party observations, we found that women partners were perceived as higher in warmth (*b* = −0.30, *t*(1,816) = −5.11, *P* < 0.001) compared to men. We also found, contrary to stereotypes, that women were perceived as higher in competence than men (*b* = −0.15, *t*(1,742) = −3.13, *P* = 0.002). Therefore, women partners were rated higher on both stereotype dimensions.

##### Subjective value.

Replicating the results of Study 2, we found participants reported greater liking of women negotiation partners (*b* = −0.29, *t*(1,816) = −4.55, *P* < 0.001).

##### Satisfaction and desire for future negotiations.

We found that participants who were partnered with women reported greater satisfaction (*b* = −0.28, *t*(1,856) = −4.14, *P* < 0.001) and a greater desire for future negotiations (*b* = −0.38, *t*(1,857) = −4.99, *P* < 0.001) compared to those partnered with men.

##### Exploratory mediation analyses.

There was a significant indirect effect of partner gender on satisfaction and desire for future negotiations, through liking (satisfaction: *b*indirect = −0.250, 95% CI [−0.359, -0.153], *z* = −4.62, *P* < 0.001; desire for future negotiations: *b*indirect = −0.296, 95% CI [−0.425, −0.181], *z* = −4.64, *P* < 0.001). Once again, those negotiating with women partners had greater satisfaction and a greater desire for future negotiations, following improved subjective value (liking).

#### Gender information condition (test of actual vs. perceived gender differences).

##### Stereotype content.

We found that gender predicted warmth, such that women partners were perceived as higher in warmth (*b* = −0.32, *t*(1,798) = −5.34, *P* < 0.001). We also found that self-reported gender still predicted higher competence for women than men partners (*b* = −0.16, *t*(1,744) = −3.22, *P* = 0.001). We found no effect of perceived gender on warmth or competence (warmth: *b* = −0.12, *t*(1,807) = −1.94, *P* = 0.052; competence: *b* = 0.02, *t*(1,752) = 0.40, *P* = 0.693). Once again, women partners were rated higher on both stereotype dimensions.

##### Subjective value.

Consistent with the control condition and the results of Study 2, we found participants report greater liking (*b* = −0.32, *t*(1,826) = −4.82, *P* < 0.001) with women partners compared to those partnered with men. We found no effect of perceived gender on liking (*b* = −0.07, *t*(1,835) = −1.03, *P* = 0.302).

##### Satisfaction and desire for future negotiations.

Also consistent with the control condition, participants reported greater satisfaction (*b* = −0.35, *t*(1,833) = −4.98, *P* < 0.001) and a greater desire for future negotiations (*b* = −0.34, *t*(1,853) = −4.54, *P* < 0.001) with women partners compared to those partnered with men. We found no effect of perceived gender on satisfaction (*b* = −0.07, *t*(1,842) = −1.03, *P* = 0.303) or the desire for future negotiations (*b* = −0.08, *t*(1,861) = −1.01, *P* = 0.313).

##### Exploratory mediation analyses.

Again, there was a significant indirect effect of partner gender on satisfaction and desire for future negotiations, through liking (satisfaction: *b*indirect = −0.270, 95% CI [−0.378, -0.167], *z* = −4.96, *P* < 0.001; desire for future negotiations: *b*indirect = −0.313, 95% *CI* [−0.439, −0.194], *z* = −4.96, *P* < 0.001). Once again, those negotiating with women partners reported greater satisfaction and a greater desire for future negotiations, following improved subjective value (liking).

This study replicates the women’s advantage in subjective value observed in Studies 1 and 2 and suggests that women’s behaviors drive these effects, beyond stereotypes. However, we acknowledge that the null effect of perceived gender in this study does not prove that stereotypes have no impact or effect on these perceptions.

### Study 4.

We previously found evidence of a preference for women negotiators that persisted even when negotiators remained anonymous to one another and that these gender differences in subjective value stemmed from actual behavioral differences. The goal of this study was to determine whether this effect generalizes to an established measure of subjective value (15) and whether the nature of the future negotiation matters. In the prior studies, we did not specify the nature of the future negotiation. Perhaps people prefer women negotiators because they believe they can extract more value (in a competitive negotiation), or only when the negotiation requires cooperation to achieve an optimal outcome (in a cooperative negotiation). Further, does the preference for women partners extend to wanting them as teammates in a future negotiation? To address these questions, we replicated the control condition in Study 3, varying the subjective value measure to align with prior literature and the nature of the future negotiation interaction.

We recruited 400 U.S. participants from Prolific and 400 participants completed this experiment. Per our preregistration, we excluded seven participants who indicated they did not read each conversation carefully, and an additional 22 participants who failed the attention check. The final sample consisted of 371 participants (181 women, 181 men, 6 nonbinary individuals, 1 something not listed, and 2 who preferred not to answer). All the remaining demographic information is reported in the *Materials and Methods*.

Participants were given a link to complete the survey, in which they read recreated chat negotiations using the same dialogues from the online negotiation study (Study 2). The procedure was the same as the control condition in Study 3, such that each participant was shown a random subset of five of the 100 possible conversations. Participants were randomly assigned to imagine they were one of the agents in the conversation. Participants read conversations with no gender information. After reading each conversation, participants provided their perceptions of their partner and the negotiation.

#### Subjective value.

Replicating the prior results, we found participants reported greater subjective value with women negotiation partners (*b* = −0.37, *t*(1,810) = −6.14, *P* < 0.001). Examining each individual factor of the subjective value inventory, we found that women partners were rated higher than men across all measures (feelings about the relationship: *b* = −0.49, *t*(1,823) = −6.77, *P* < 0.001; feelings about the process: *b* = −0.42, *t*(1,829) = −6.19, *P* < 0.001; feelings about the self: *b* = −0.24, *t*(1,753) = −4.54, *P* < 0.001; feelings about the outcome: *b* = −0.36, *t*(1,813) = −5.57, *P* < 0.001).

#### Desire for future negotiations.

**Fig. 3.**
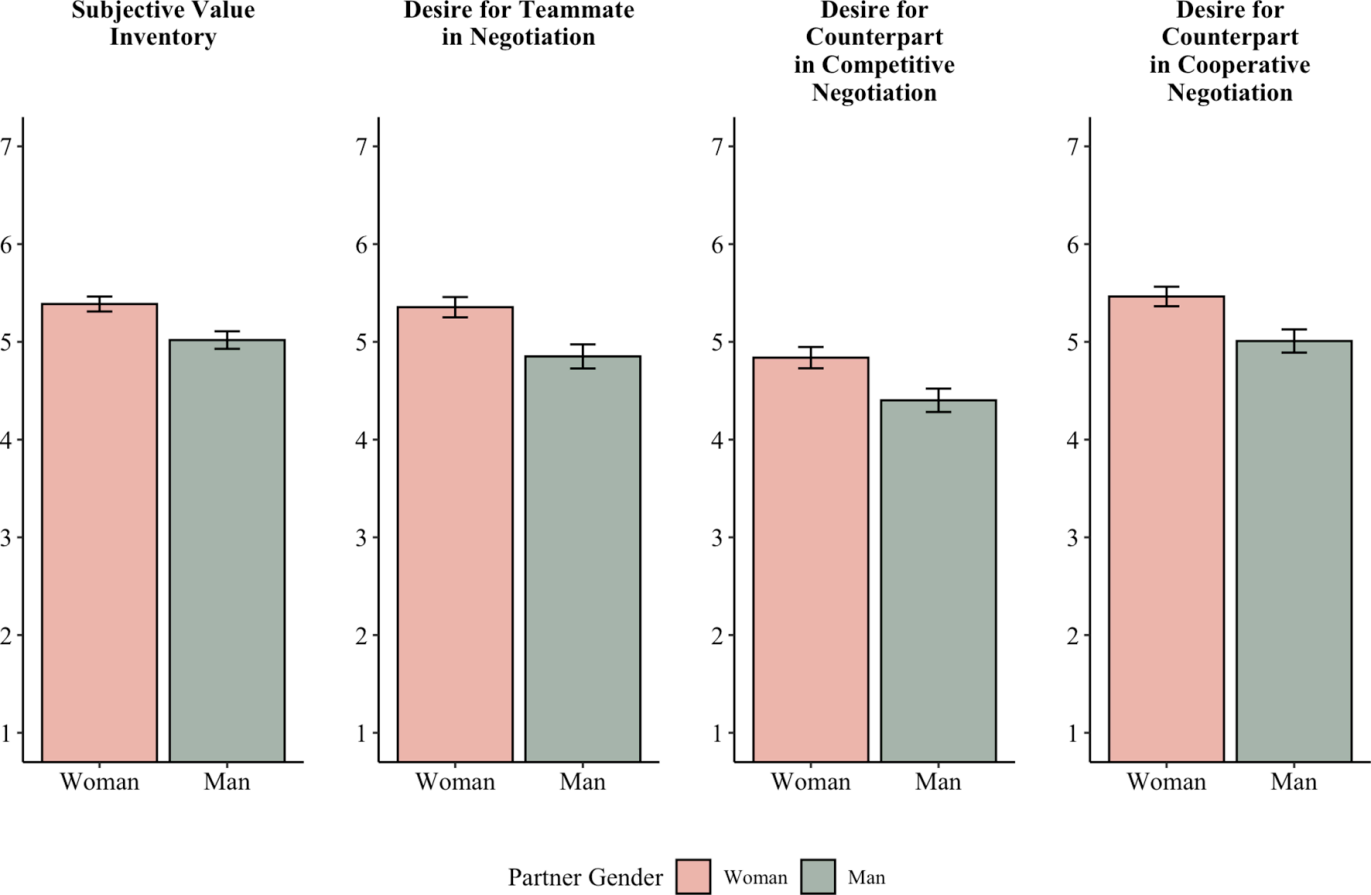
Women partners were rated significantly higher than men partners in subjective value and desire for future negotiations across all future negotiation types (Study 4). Note. (*N* = 371) The error bars are 95% CI.

We found that participants who were partnered with women reported a greater desire for them to be their teammate (*b* = −0.50, *t*(1,840) = −6.13, *P* < 0.001) and their counterpart in competitive (*b* = −0.41, *t*(1,741) = −5.20, *P* < 0.001) and cooperative (*b* = −0.46, *t*(1,825) = −5.79, *P* < 0.001) negotiations compared to those partnered with men. Fig. 3 displays these ratings by partner gender.

#### Preregistered mediation analyses.

There was a significant indirect effect of partner gender on all desire for future negotiation measures, through subjective value (teammate: *b*indirect = −0.44, 95% CI [−0.58, −0.31], *z* = −6.46, *P* < 0.001; competitive: *b*indirect = −0.33, *95%* CI [−0.43, −0.24], z = −6.50, *P* < 0.001; cooperative: *b*indirect = −0.39, 95% CI [−0.52, −0.28], z = −6.47, *P* < 0.001). Once again, negotiating with women partners led to a greater desire for future negotiations, regardless of the nature of the future interaction, following improved subjective value.

We then ran the mediation model with each individual factor of the subjective value inventory as parallel mediators, on teammate (relationship: *b*indirect = −0.34, 95% *CI* [−0.43, −0.25], *z* = −7.29, *P* < 0.001, process: *b*indirect = −0.14, 95% *CI* [−0.20, −0.09], *z* = −5.10, *P* < 0.001; self: *b*indirect = 0.01, 95% *CI* [0.00, 0.03], *z* = 1.68, *P* = 0.093; outcome: *b*indirect = −0.03, 95% CI [−0.07, −0.01], *z* = −2.34, *P* = 0.019), competitive (relationship: *b*indirect = −0.18, 95% CI [−0.24, −0.13], *z* = −6.09, *P* < 0.001, process: *b*indirect = −0.16, 95% CI [−0.23, −0.10], *z* = −4.46, *P* < 0.001; self: *b*indirect = 0.01, 95% CI [−0.01, 0.03], *z* = 0.47, *P* = 0.636; outcome: *b*indirect = −0.03, 95% CI [−0.08, 0.01], *z* = −1.41, *P* = 0.159), and cooperative (relationship: *b*indirect = −0.25, 95% CI [−0.32, −0.19], *z* = −7.12, *P* < 0.001, process: *b*indirect = −0.17, 95% CI [−0.25, −0.12], *z* = −5.17, *P* < 0.001; self: *b*indirect = 0.01, 95% CI [−0.00, 0.03], *z* = 1.38, *P* = 0.168; outcome: *b*indirect = −0.03, 95% CI [−0.06, 0.00], *z* = −1.66, *P* = 0.097) future negotiations. As preregistered, we found that participants’ feelings about the relationship and about the process, which both concern perceptions of negotiation counterparts, mediated all three future negotiation intention measures, whereas feelings about the self and the outcome did not.

### Study 5.

Building on the previous studies demonstrating gender differences in subjective value favoring women, the evidence suggests these disparities stem from behavioral differences rather than mere stereotypes. To identify the specific behavioral patterns underlying these differences, we used an inductive analytical approach. This was an exploratory examination of potential gender differences in behaviors.

We analyzed negotiation transcripts from Study 2 using established coding schemes, implemented through AI-assisted coding software designed for negotiation analysis (68). The model categorized behaviors into twelve distinct codes: provide information, miscellaneous off-task information, miscellaneous on-task information, accept offer, multiissue offer, reject offer, single-issue offer, process comment, question, negative reaction, positive reaction, and substantiation, or no code. Each speaking turn served as the unit of analysis, with the model assigning appropriate behavioral codes to each unit.

We found that women partners were significantly more likely to accept offers (*b* = −0.18, *z* = −3.39, *P* < 0.001) and make multiissue offers (*b* = −0.08, *z* = −1.97, *P* = 0.049). In contrast, men partners were significantly more likely to share information (*b* = 0.17, *z* = 3.35, *P* < 0.001) and provide miscellaneous on-task information (*b* = 0.32, *z* = 3.47, *P* < 0.001). We found no significant differences for the remaining codes: question, positive reaction, negative reaction, substantiation, single issue offers, reject offers, process comments, and miscellaneous off-task information (all *Ps* > 0.10).

#### Mediation.

We then conducted a parallel mediation model to determine whether these partner-based gender differences in negotiation behaviors (accepting offers, multiissue offers, providing information, and miscellaneous on-task information) could explain the gender gap in the subjective value outcome of partner liking.

There was a significant indirect effect of partner gender on subjective value through accepting offers (*b*indirect = −0.023, 95% CI [−0.038, −0.010], *z* = −3.04, *P* = 0.002). Accepting offers was the only mediator that remained significant after controlling for participant gender and points, and after including a random intercept for the dyad.

There was also a significant indirect effect of partner gender on liking through providing information (*b*indirect = 0.010, 95% CI [0.003, 0.021], *z* = 2.17, *P* = 0.030), such that men provided more information, which was correlated with greater liking. The indirect effects through multiissue offers on liking, and through miscellaneous on-task information on liking, were both not significant (multiissue offers: *b*indirect = 0.007, 95% CI [−0.00, 0.015], *z* = 1.60, *P* = 0.109; miscellaneous on-task information: *b*indirect = 0.005, 95% CI [−0.003, 0.015], *z* = 1.22, *P* = 0.224).

We then used a serial mediation model to test the full model with offer acceptance. We examined whether the effect of partner gender on satisfaction was mediated by offer acceptance and liking. We found a significant total indirect effect (*b*indirect = −0.081, 95% CI [−0.145, −0.023]). We also found the sequential path through offer acceptance and liking was significant (*b*indirect = −0.016, 95% CI [−0.027, −0.006]). We found the results remain significant, controlling for participant gender and points. The model is shown in *SI Appendix*, Fig. S4. The method we used does not currently support multilevel serial mediation, which is why we did not examine the results with a random intercept in this model.

In summary, we found that women were more likely to accept offers, which is the most robust effect in explaining the gender gap in their partner’s subjective value and satisfaction, and without compromising women’s economic outcomes. We found evidence of a pathway through which women were more likely to accept offers, which led their partners to have a greater subjective value, contributing to greater satisfaction with the negotiation. This result is consistent with prior work, which has illuminated the effect of concessions on partners’ perceptions of the negotiation and suggested that concessions by a counterpart can lead to greater perceptions of fairness and feeling heard, which contribute to more positive evaluations of the counterpart and a greater desire to negotiate with the same counterpart again (69). More recent research has further emphasized that yielding behavior (i.e., accepting offers) generates greater subjective value for counterparts (70). We note that this coding methodology categorizes behavior into codes previously identified in the negotiation literature, though there may be other behavioral differences between men and women not captured that future work might explore.

### Simulation.

To conceptualize the significance of a partner gender difference in desire for future negotiations, we conducted a simulation based on Study 1’s observed difference with the following assumptions: everyone participates in at least one negotiation to start, and after each negotiation, there is a probability, *P*, that the counterpart wants to negotiate again. This probability, *P*, is constant over time and the only variation between men and women. Therefore, if the counterpart does not want to negotiate again with the probability 1 – *P*, then the sequence stops. Based on these assumptions, we ran a Monte Carlo simulation (71) assigning the probabilities, *P*_women_ and *P_men_*, for women and men, respectively, that their partner wants to negotiate with them again based on Study 1’s results (*P_women_* = 0.944, *P_men_* = 0.919). For each simulation, our model randomly selected 5,000 outcomes for women and 5,000 outcomes for men from a geometric distribution to determine the number of negotiation opportunities until the sequence stopped. We ran this simulation 2,000 times and computed the ratio of the mean number of opportunities to negotiate for women to that for men in each simulation. We found that on average, women would have 44.56 percent more negotiation opportunities than men over time (*M* = 1.45, 95% CI [1.39, 1.50], *Mdn* = 1.44, 95% CI [1.33, 1.63]). *SI Appendix*, Fig. S5 displays the distribution of ratios of the mean number of negotiations for women and men in the Monte Carlo simulation. To further quantify the cumulative impact of repeated negotiations, we emulated research that has simulated the effects of exercising negotiation opportunities (72, 73) and assign a value to each negotiation. Let us imagine a consultant receives a $10,000 bonus for every client project they secure for their firm. Alternatively, a salesperson who receives a 10 percent bonus for every sale worth $100,000. Assuming men and women achieve similar negotiated outcomes in either scenario, the return per additional negotiation is $10,000. Using the average number of negotiations for women (*M* = 17.85, 95% CI [17.35, 18.35]) and men (*M* = 12.35, 95% CI [12.02, 12.68]), from the simulation, women and men would net $178,451 and $123,501, respectively, creating an average gender gap of $54,950.

## Discussion

Our findings reveal women’s advantage in subjective value during negotiations, where they are more likely to accept offers, leading to greater liking, satisfaction, and a desire to negotiate with them again.

In Study 1, we analyzed classroom negotiation feedback data and found that women partners received higher ratings of subjective value in terms of building trust, demonstrating fairness, satisfying their partner’s needs, expanding the pie, communicating effectively, listening attentively, and overall effectiveness. These relational strengths, particularly trust building, fairness, and attention to partner interests, directly contributed to participants’ greater desire to negotiate with women again.

Study 2 examined partner-based gender effects in anonymous online negotiations. Even when negotiators’ gender remained unidentified, women partners were more liked, which predicted greater satisfaction, independent of economic outcomes. A pretest confirmed that third parties could not infer gender from these online negotiations, validating their anonymity and supporting the notion that partner-based gender effects on subjective value reflect behavioral differences in negotiation strategy rather than mere stereotypes.

In Study 3, we applied the stereotype content model (41, 42) to examine negotiator perceptions. Women partners were rated higher on both warmth and competence, defying the stereotype that women are relatively low in competence, and were also better liked, which in turn fostered greater satisfaction and a stronger desire to negotiate with them again. These results held when perceived gender was randomly assigned. In our control condition, and in Studies 2 and 4, we found evidence of women’s advantage in subjective value even when participants are unaware of their counterpart’s gender. When partner gender is unknown, women need not surpass men in subjective value in order to achieve equivalent economic outcomes. Moreover, manipulating perceived gender did not alter partner perceptions, suggesting that participants held similar expectations for women and men in this context. Had expectations differed, women who violated gendered norms might have incurred the social penalties (i.e., backlash) documented in vignette studies that held the negotiation exchange constant while varying target gender (5).

In Study 4, we found that women’s advantage in subjective value generalizes to a validated, multidimensional scale measuring subjective value, and that individuals prefer women in all future negotiation scenarios, be it as teammates, or as counterparts in both competitive and cooperative negotiations.

Our final study examined actual negotiation behaviors and found that women were more likely to accept offers, which contributed to the observed gender gap in partner liking and subsequently, satisfaction. While early 2000 s research suggested that women’s relational concerns led them to accept offers without negotiating (resulting in worse outcomes), our findings indicate that women now achieve equivalent economic outcomes while generating superior subjective value for their negotiating partners through the strategic acceptance of offers.

This research addresses recent calls in the literature to further explore gender dynamics and perceptions in negotiations (74). Our findings align with emerging research that challenges previous assumptions about gender differences and recognizes women’s negotiation strengths (39, 40). Kray et al. (2023) demonstrated that women now initiate negotiations more frequently than men, contradicting decades of opposing assertions. Similarly, Ma et al. (39) and Bowles et al. (75) showed that women avoid impasses and outperform men due to their relational orientation. Emerging work in a market setting also shows, using a field experiment, the cumulative positive economic impact of customers preferring to approach women more than men in markets (76). These findings reflect a broader trend of women excelling in domains previously considered masculine (77).

Importantly, our research demonstrates that women achieve superior subjective value without sacrificing economic performance; there were no gender differences in tangible negotiation outcomes.

### Limitations and Future Directions.

Negotiation researchers have long moved beyond the oversimplified assertion, from the 1970s and early 1980s, that negotiator gender is a stable predictor of behavior (10). Our results may reflect a main gender effect moderated by various contextual behaviors that warrant future exploration. Further, while this research demonstrates the advantages of accepting offers, this may be one of many factors contributing to women’s advantage in subjective value. Critically, future research should also explore this in real-world negotiations to increase the generalizability of these findings, as well as varying the stakes of the negotiation.

One limitation of our studies is that negotiations occurred in low-ambiguity settings where the appropriateness of negotiating was clear; participants were either enrolled in negotiation courses or voluntarily participated in experiments. Situational ambiguity may moderate gender effects on subjective value, as it moderates economic outcomes. Bowles et al. found that women have worse economic outcomes than men in ambiguous situations (78). Therefore, future research is needed to determine whether women’s advantage in subjective value extends to situations in which the expectation to negotiate is not as explicit.

Additionally, prior research has warned against the potential downsides of excessive communality (51). When both negotiators prioritize relationships over individual interests, economic value may be lost. Prioritizing relationships could lead negotiators to satisfice, resulting in diminished economic outcomes (75). However, our findings suggest that women’s advantage in creating subjective value does not compromise their economic performance.

### Implications for Negotiation Theory and Practice.

Negotiations occur at pivotal life moments, making this an important area for targeted research (4). Prior work has documented a tendency to stereotype men as assertive and competitive negotiators, while viewing women as agreeable and cooperative bargainers (79). Many attributes associated with effective negotiations have been perceived as masculine, while characteristics defining ineffective negotiation have been labeled feminine (80).

Responding to calls to update our understanding of successful negotiators (31), we examined whether women’s recognized strengths, such as cooperativeness and trustworthiness, yield greater subjective value. This work challenges the stereotype that men are inherently superior negotiators. By highlighting the importance of traditionally feminine traits in achieving positive subjective value, we hope to reshape perceptions of negotiator effectiveness (2, 81). To reach this ideal requires continued effort to dispel myths that may prevent women from recognizing and leveraging their demonstrated strengths in negotiation contexts.

## Materials and Methods

Our original data and materials are available at: https://osf.io/52qwc/?view_only=a10eade50990460ba7b0c5e0b7401ef3. The studies were reviewed and approved by the IRB at each US university study site (UC Berkeley #2023-04-16259 and Cornell #IRB0148978). Informed consent was obtained from all participants, with the exception of Study 1. Study 1 data were originally collected for regular educational activities, not research, and were then deidentified for research purposes; therefore, they should pose minimal risk from secondary analysis.

Previously published data were used for this work (64).

### Study 1.

#### Experimental design.

This was our initial test of whether there is a gender gap in subjective value in negotiations. We used an archival dataset of existing measures from a full-time MBA negotiation course. The dataset includes over 2,000 observations from 231 students. In this course, students completed face-to-face negotiation role-playing exercises followed by postnegotiation online surveys every week. Students received credit for completing feedback as a homework assignment. Students were randomly assigned their negotiation roles and negotiation partners. Students debriefed the negotiations after they took place and before completing the survey. In the postnegotiation surveys, students evaluated themselves and their partners using the same measures and provided open-ended feedback on their strengths and areas for improvement. Students primarily provided feedback to their negotiation counterparts but may also have evaluated other group members.

#### Measures.

##### Subjective value.

Participants evaluated their partners on the extent from 1 (*Not at all*) to 7 (*Very Much)* they: were effective in building *trust*, *fair*, concerned with *satisfying their partner’s interests*, looked after *their own interests*, acted very *competitively*, tried to create value and *expand the pie*, were an active and effective *communicator*, were an active and effective *listener*, and effective *overall*.

We also collected self-evaluations and found significant participant gender differences in self-evaluations for competitiveness and listening, such that men rated themselves higher in competitiveness but lower in listening. *SI Appendix*, Tables S5–S8 contain the results.

##### Desire for future negotiations.

Participants also responded [Yes/No] to “Given the way your partner negotiated, would you want to work with your partner again?”

##### Economic outcomes.

For the first negotiations, the students participated in negotiations with calculated economic outcomes (Yerba Mate, New Bike, and Internship). For the following negotiations: Salary, Tech Now, Grand Strand, and Bullard Houses, Research Assistants were trained to independently code negotiation outcomes from the student reported agreements.

*Distributive outcomes* for dyads that reached an agreement were the summed monetary agreements of each negotiation. For instance, salary, bonus, stock, or sale price. We then winsorized outcomes at the bottom and top one percent to account for outliers or for student errors inputting agreements that may have resulted in outliers. We then z-scored the points outcomes within negotiations and used this as a measure of comparative economic outcome. For Yerba Mate, Salary, Tech Now, Grand Strand, and Bullard Houses, the standardized scores were reversed for students in the roles of buyer, boss, and manager, as higher economic outcomes in the negotiation indicate worse performance in these roles.

*Integrative outcomes* were coded by Research Assistants based on prior negotiation research. Agreements were coded “0” if dyads did not reach an agreement, “1” if dyads reached a nonintegrative agreement, and “2” if they reached an integrative agreement (58, 82). New Bike and Intern negotiations had specified integrative issues; therefore, outcomes were coded as “0” if no agreement was reached and “2” if an agreement was reached.

#### Statistical analysis.

We tested the effect of participant gender and partner gender on dependent measures. We included a random intercept for the student/participant who completed the survey. *SI Appendix*, Tables S1 and S2 include the full results. We also found that men rated their partners lower for communicating (*b* = −0.19, *t*(233) = −2.45, *P* = 0.015), listening (*b* = −0.17, *t*(232) = −2.31, *P* = 0.022), and overall (*b* = −0.20, *t*(229) = −2.79, *P* = 0.006). Participant Gender x Partner Gender interaction did not predict any of the dependent measures, but is included in *SI Appendix*, Tables S3 and S4 for comprehensiveness. For the desire for future negotiations, we ran a multilevel logistic regression with a random intercept for the student/participant who completed the survey. The effect of participant gender on desire for future negotiations was not significant (*b* = 0.03, *P* = 0.893).

For the negotiations with measurable economic outcomes, we regressed participant gender and partner gender on the outcome measure and included a random intercept for the student/participant who completed the survey. We winsorized z-scored distributive outcomes. To assess integrative outcomes, we used a chi-square test.

Last, we conducted a mediation model, with ratings of building trust, being fair, satisfying partner interests, expanding the pie, communication, listening, and overall effectiveness as mediators, and accounting for the binary-dependent measure. We included participant gender as a covariate and included a random intercept for the student/participant who completed the survey.

For the specification curve analysis, we consider the results of all subjective value measures, varying the model (linear or multilevel) and the inclusion of covariates (participant gender). *SI Appendix*, Figs. S1 *A* and *B* and S2 *A* and *B* contain the specification curve analysis: *SI Appendix*, Fig. S1 *A* and *B* corresponds with linear regression results, and *SI Appendix*, Fig. S2 *A* and *B* corresponds with results using a multilevel model. A figures contain the specification curves highlighting nonsignificant (in gray) and positive (in blue) effects. No negative effects are reported. B figures include vertically aligned points indicating the independent variable (partner gender), the dependent variable, the model type, and whether covariates were included in each model.

### Study 2.

#### Experimental design.

The goal of this study was twofold: replicate the gender gap in subjective value, and explore whether this effect is due to differences in behavior or perceptions. We used an archival dataset from previously published research, available at https://huggingface.co/datasets/casino (64). The authors recruited participants from Amazon Mechanical Turk. The sample was primarily White Americans (*N* = 625), and participants also identified as Black or African American, Asian American, Hispanic or Latino, Multiracial, Alaska Native or Native American, Native Hawaiian or Other Pacific Islander, and Other (open-ended). However, the original authors did not report all individual ethnic information, and the data do not include identifiers for each participant across dyads, so we are not able to report exact ethnic composition. The average age was *M* = 36.97 y, SD = 10.81. They first conducted a presurvey of participants’ demographic information and personality measures. Chawla et al. (64) report all the measures collected. Next, all participants watched a video tutorial before their negotiation, which showed an example of a negotiation to encourage best practices and how to use the negotiation platform. Next, they participated in an online negotiation, where they were randomly paired and could communicate via chat to devise a deal. Although the negotiation was over fictitious campfire resources, participants were incentivized via a performance-based bonus of 8.33 cents per point, and each participant could receive a maximum of 36 points. Once they agreed on a deal or reached an impasse, they completed a postsurvey questionnaire. In the postnegotiation survey, students completed measures about their negotiation experience.

#### Measures.

##### Subjective value.

###### Liking.

Participants responded to “How much do you like your opponent?” from 1 (*Extremely dislike*) to 5 (*Extremely like*).

##### Satisfaction.

Participants responded to “How satisfied are you with the negotiation outcome?” from 1 (*Extremely dissatisfied*) to 5 (*Extremely satisfied*).

##### Economic outcome.

Participants received points based on their final deal. If they arrived at an impasse, each participant received five points. Participants could earn a maximum of 36 points individually but jointly could earn either 36, 39, or 42 points (depending on the random assignment of points to resources). Each campfire resource was randomly assigned a value of high (five points per item), medium (four points per item), or low (three points per item) for each participant, and they negotiated the division of three items for each resource. Due to randomization, each dyad’s negotiation varied in the extent to which it was distributive (maximum 36 points) or integrative (maximum 42 points). Of the dyads, 257 had distributive, 378 had mixed motive, and 395 had integrative negotiations.

#### Statistical analysis.

We tested the effect of participant and partner gender on partner liking and satisfaction. We included a random intercept for the negotiation dyad. We also found that men liked their counterparts less than women (*b* = −0.19, *t*(1,882) = −3.99, *P* < 0.001), even when controlling for points (*b* = −0.19, *t*(1,921) = −4.11, *P* < 0.001), and that men were less satisfied than women (*b* = −0.16, *t*(2,017) = −3.55, *P* < 0.001). Participant Gender × Partner Gender interaction did not predict any dependent measures. For the economic outcome, we regressed participant gender and partner gender on points. Participant Gender × Partner Gender interaction did not predict points or joint points.

For the mediation analyses, we used a mediation model with partner gender as a predictor, participant gender as a covariate, and a random intercept for the negotiation dyad. The indirect effect is also significant when we include points as a covariate (*b*indirect = −0.062, 95% CI [−0.115, −0.011], *z* = −2.33, *P* = 0.020).

### Study 2 Pretest.

#### Experimental design.

In this pretest, we aimed to demonstrate that participants were unable to infer gender in anonymous negotiations. We recruited undergraduates at a large research university who completed the survey for course credit. Thus, the sample size was determined by course enrollment. All participants in the study completed the survey during the Spring Semester of 2023. Following the preregistration, we excluded 65 participants who indicated they did not read the survey carefully. The final sample consisted of 180 participants (97 women, 81 men, and 2 nonbinary individuals). Ethnic composition was as follows: 63 East Asian/East Asian American, 48 White/Caucasian, 34 South Asian/South Asian American, 29 Southeast Asian/Southeast Asian American, 21 Latino/Hispanic American, 9 Black/African American, 6 Middle Eastern/Arab American, 2 Native/American Indian, and 2 as a group not listed. Participants could select multiple ethnicities. The average age was M = 21.2 y, SD = 2.62. Participants were given a link to complete the survey, in which they read recreated chat negotiations using the dialogues from real participants in Study 2. The procedure was based on prior work, in which participants try to infer and indicate the gender of someone online when gender information is not explicitly provided, for instance, in online marketplace profiles (65, 66). We randomly selected a subset of 100 dyads and displayed their conversations to participants. All the agents in the subset of conversations self-identified as either a woman or a man. Each participant was shown five of the 100 possible conversations. For an example of a conversation, see *SI Appendix*, Fig. S3.

#### Measures.

##### Gender inference.

Participants were asked, “Please evaluate which gender you believe each agent is” and the response options were Woman, Man, or Cannot discern.

#### Statistical analysis.

For analyses, we used a binomial *t* test, comparing participant accuracy to 50 percent accuracy, or chance. Accuracy was a binary measure, coded as 0 if participants incorrectly inferred the agent’s gender, and “1” if participants were correct. Cannot discern was coded as “0,” or incorrect. In exploration, we conducted a chi-square test to examine whether there was an association between the inferred gender and accuracy.

### Study 3.

#### Experimental design.

We previously demonstrated women’s advantage in subjective value and that these are due to behavioral differences. However, we wanted to test whether these behavioral differences were stronger than stereotypes. We recruited 800 U.S. participants from Prolific and 803 participants completed this experiment. In accordance with the preregistration, we excluded 30 participants who indicated they did not read each conversation carefully. The final sample consisted of 773 participants (372 women, 383 men, 13 nonbinary individuals, and five who preferred not to answer). Ethnic composition was as follows: 498 White/Caucasian, 136 Black/African American, 81 Hispanic/Latino, 77 East Asian/East Asian American, 28 Southeast Asian/Southeast Asian American, 17 South Asian/South Asian American, and nine who identified as something not listed. Participants could indicate multiple ethnicities. The average age was *M* = 38.9 y old, SD = 12.8.

#### Procedure.

Participants were given a link to complete the survey, in which they read recreated chat negotiations using the same dialogues from the online negotiation study. The procedure was similar to the gender inference study (Study 2 pretest), except we manipulated the gender information provided. Participants were randomly assigned to imagine they were one of the agents in the conversation. The other agent in the conversation was randomly assigned to be a woman or a man, or the control condition (in which no gender information is provided). We used the same random subset of 100 conversations from the Study 2 pretest to display to participants, and each participant was shown five of the 100 possible conversations. Participants either read five conversations with no identifying gender information or read five conversations in which the other agent was randomly assigned a gender (between subjects). After reading each conversation, participants provided their perceptions of their partner and the negotiation.

#### Measures.

##### Stereotypes.

*Warmth*. Participants evaluated agent warmth using a composite of six items (42): “In your opinion, how friendly is [target]?”, “… how well-intentioned is [target],” “… how trustworthy is [target],” “… how warm is [target],” “… how good-natured is [target],” “… how sincere is [target],” from 1 (*Not at all*) to 7 (*Extremely)* (α = 0.96).

***Competence***. Participants evaluated agent competence using a composite of six items (42): “In your opinion, how competent is [target]?”, “… how confident is [target],” “… how capable is [target],” “… how efficient is [target],” “… how intelligent is [target],” “… how skillful is [target],” from 1 (*Not at all*) to 7 (*Extremely)* (α = 0.95).

##### Subjective value.

*Liking*. Participants responded to “How much would you like [target]?” from 1 (*Extremely dislike*) to 7 (*Extremely like*).

##### Satisfaction.

Participants responded to “How satisfied would you be with the negotiation outcome?” from 1 (*Extremely dissatisfied*) to 7 (*Extremely satisfied*).

##### Desire for future negotiations.

Participants responded to “How much would you like to negotiate with [target] in the future?” from 1 (*Extremely dislike*) to 7 (*Extremely like*).

#### Statistical analysis.

We analyzed the data separately for each condition as preregistered. We conducted multilevel model regressions predicting perceived warmth and competence from agents’ self-reported gender in the control condition. We also added the variable for gender displayed in the gender information condition, for which both gender variables were included in the models simultaneously. We also conducted multilevel model regressions predicting third-party ratings of liking, satisfaction, and desire for future negotiations from agents’ self-reported gender and the gender displayed to participants in the gender information condition. We included random intercepts for participants. Participant gender did not significantly moderate these results. For participant gender analyses, we excluded participants who responded “Prefer not to answer” to their gender.

For the mediation analyses in the control condition, we used a mediation model with partner gender as a predictor, liking as the mediator, and satisfaction and desire for future negotiations as dependent measures, and included a random intercept for participants. For the mediation analyses in the gender information condition, we used a mediation model with partner gender as a predictor, liking as the mediator, and satisfaction and desire for future negotiations as dependent measures, and included a random intercept for participants. The indirect effects are also significant when perceived gender is included as a covariate (satisfaction: *b*indirect = −0.276, 95% CI [−0.407, −0.156], *z* = −4.20, *P* < 0.001; desire for future negotiations: *b*indirect = −0.320, 95% CI [−0.472, −0.181], *z* = −4.20, *P* < 0.001).

***Control condition***. We also examined the effect of participant gender, as preregistered, and found that participant gender did not significantly predict or moderate these results. For these analyses, we removed participants who responded “Prefer not to answer” when asked to report their gender.

***Gender information condition***. Participant gender significantly predicted warmth (*P* = 0.038) and desire for future interaction (*P* = 0.017), such that women participants gave lower ratings across these dimensions. Participant gender did not predict competence (*P* = 0.278), liking (*P* =0.066), or satisfaction (*P* = 0.087). The main effects of partner gender remained significant when participant gender was included in the model, and participant gender did not moderate the results. For these analyses, we removed participants who responded “Prefer not to answer” when asked to report their gender.

### Study 4.

#### Experimental design.

We previously demonstrated women’s advantage in subjective value and that these are due to behavioral differences, and that they are stronger than stereotypes. Next, we tested the generalizability of this result. We recruited 400 U.S. participants from Prolific and 400 participants completed this experiment. Per our preregistration, we excluded seven participants who indicated they did not read each conversation carefully, and an additional 22 participants who failed the attention check. The final sample consisted of 371 participants (181 women, 181 men, 6 nonbinary individuals, 1 something not listed, and 2 who preferred not to answer). Ethnic composition was as follows: 296 White/Caucasian, 36 Latino/Hispanic American, 33 Black/African American, 12 East Asian/East Asian American, 6 Southeast Asian/Southeast Asian American, 5 South Asian/South Asian American, 2 Middle Eastern/Arab American, and 2 Native/American Indian. Participants could indicate multiple ethnicities. The average age was *M* = 43.5 y, SD = 13.4.

##### Procedure.

Participants were given a link to complete the survey, in which they read reconstructed chat negotiations using the same dialogues from the online negotiation study. The procedure was similar to Study 3, except we did not manipulate the gender information provided (we replicated the control condition only). Participants were randomly assigned to imagine they were one of the agents in the conversation and no gender information was provided about their counterpart. We used the same random subset of 100 conversations from Study 3 to display to participants, and each participant was shown five of the 100 possible conversations. Participants read five conversations with no identifying gender information. After reading each conversation, participants provided their perceptions of their partner and the negotiation.

#### Measures.

##### Subjective value.

*Subjective value inventory*. Participants evaluated subjective value using a composite of thirteen items (16): “The negotiation built a good foundation for a future relationship with my counterpart,” “The negotiation made me trust my counterpart,” “Overall, my counterpart made a positive impression on me,” “My counterpart considered my wishes, opinions, and needs,” “The negotiation process was fair,” “I am satisfied with the ease (or difficulty) of reaching an agreement,” “This negotiation made me feel more competent as a negotiator,” “This negotiation positively impacted my self-image or impression of myself,” “I “lost face” (i.e., damaged my sense of pride) in the negotiation” (reverse-scored), “I behaved according to my own principles and values,” “I think the terms of my agreement were fair,” “I feel like I forfeited or “lost” in this negotiation” (reverse-scored),” “I am satisfied with my own outcome in this negotiation” from 1 (*Strongly disagree*) to 7 (*Strongly agree)* (α = 0.96).

The subjective value inventory can also be subdivided into four factors:

***Feelings about the relationship***. Items: “The negotiation built a good foundation for a future relationship with my counterpart,” “The negotiation made me trust my counterpart,” and “Overall, my counterpart made a positive impression on me” (α = 0.95).

***Feelings about the process.*** Items: “My counterpart considered my wishes, opinions, and needs”, “The negotiation process was fair”, and “I am satisfied with the ease (or difficulty) of reaching an agreement” (α = 0.92).

***Feelings about the self.*** Items: “This negotiation made me feel more competent as a negotiator”, “This negotiation positively impacted my self-image or impression of myself”, “I “lost face” (i.e., damaged my sense of pride) in the negotiation” (reverse-scored), and “I behaved according to my own principles and values” (α = 0.82).

***Feelings about the outcome.*** Items: “I think the terms of my agreement were fair”, “I feel like I forfeited or “lost” in this negotiation” (reverse-scored), and “I am satisfied with my own outcome in this negotiation” (α = 0.89).

##### Desire for future negotiations.

*Desire for future negotiation, teammate.* Participants responded to “How much would you like [target] to be your teammate in a future negotiation?” from 1 (Extremely dislike) to 7 (Extremely like).

***Desire for future negotiation, competitive.*** Participants responded to “How much would you like [target] to be your negotiating counterpart in a win-lose, competitive negotiation?” from 1 (Extremely dislike) to 7 (Extremely like).

***Desire for future negotiation, cooperative.*** Participants responded to “How much would you like [target] to be your negotiating counterpart in a win-win, cooperative negotiation?” from 1 (Extremely dislike) to 7 (Extremely like).

#### Statistical analysis.

We analyzed the data as preregistered. We conducted multilevel model regressions predicting perceived subjective value from agents’ self-reported gender. We also conducted multilevel model regressions predicting third-party ratings of desire for teammate, desire for future negotiations, win-lose/competitive and win-win/cooperative, from agents’ self-reported gender. We included random intercepts for participants.

For the mediation analyses, we used a mediation model with partner gender as a predictor, subjective value inventory as the mediator, and desire for future negotiations as a teammate, a counterpart in competitive and cooperative negotiations, as dependent measures, including a random intercept for participants. We also conducted the same mediation model, but with each factor of the subjective value inventory (relationship, process, self, outcome) as a parallel mediator.

### Study 5.

#### Experimental design.

To determine what behavioral differences may contribute to the gender gap in subjective value, we analyzed all transcripts from Chawla and colleagues’ data using prior negotiation coding schemes and employed an AI-assisted coding software for negotiation transcripts.

#### Measures.

We used an AI model (Model 2) from the AI Negotiation Lab at Vanderbilt University to code negotiation behaviors. The model assigned one code to each speaking turn. The model utilizes the following behavioral codes: provide information, miscellaneous off-task information, miscellaneous on-task information, accept offer, multiissue offer, reject offer, single-issue offer, process comment, question, negative reaction, positive reaction, substantiation, or no code.

#### Statistical analysis.

To test for gender differences in behaviors, we ran logistic regressions to predict each of the possible behavior codes by participant/speaker gender (i.e., Accept Offer: Yes = 1, No = 0).

For the mediation analysis, we computed the proportion of messages that were each code for each participant. We report those results in the main text. We also calculated the raw count of occurrences for each code for each participant. The model for offer acceptance as a mediator is robust, whether we use the proportion of each code per participant or the total count of occurrences of each code for each participant. The model is also robust when we include participant gender and points as covariates and include a random intercept for the negotiation dyad, except that with the raw count of occurrences, the model does not quite reach significance (*P* = 0.068). The other mediators (information, miscellaneous on-task information, and multiissue offers) were not significant when using the total count of occurrences of each code.

We then used a serial mediation model to test the full model with offer acceptance. We examined whether offer acceptance and liking mediated the effect of partner gender on satisfaction, testing the sequential path through offer acceptance and liking. We also tested the model controlling for participant gender and points. The method we used does not currently support multilevel serial mediation, so we did not examine the results with a random intercept in this model.

## Supplementary Material

Appendix 01 (PDF)

## Data Availability

Anonymized survey and behavioral data have been deposited in OSF (https://osf.io/52qwc/?view_only=a10eade50990460ba7b0c5e0b7401ef3). Previously published data were used for this work (64).
