# Supplementary material for: People prefer to negotiate with women, even when outcomes are identical and gender is unknown
Source: Proc Natl Acad Sci U S A. 2026 Jun 22;123(26):e2523202123. doi: 10.1073/pnas.2523202123 (PMC13320683; doi:10.1073/pnas.2523202123)
Supplement: Supplementary file 1 — Appendix 01 (PDF) [file pnas.2523202123.sapp.pdf]

Supporting Information for

People Prefer to Negotiate with Women, Even When Outcomes Are Identical and Gender is Unknown

Charlotte H. Townsend, Laura J. Kray, Solène Delecourt.

Correspondence to: ctownsend@cornell.edu

This PDF file includes:

Figures S1 to S5
Tables S1 to S8
Survey Materials for Study 1, Study 2 pretest, Study 3, and Study 4

Figure S1. Specification curve analysis for linear models (Study 1).

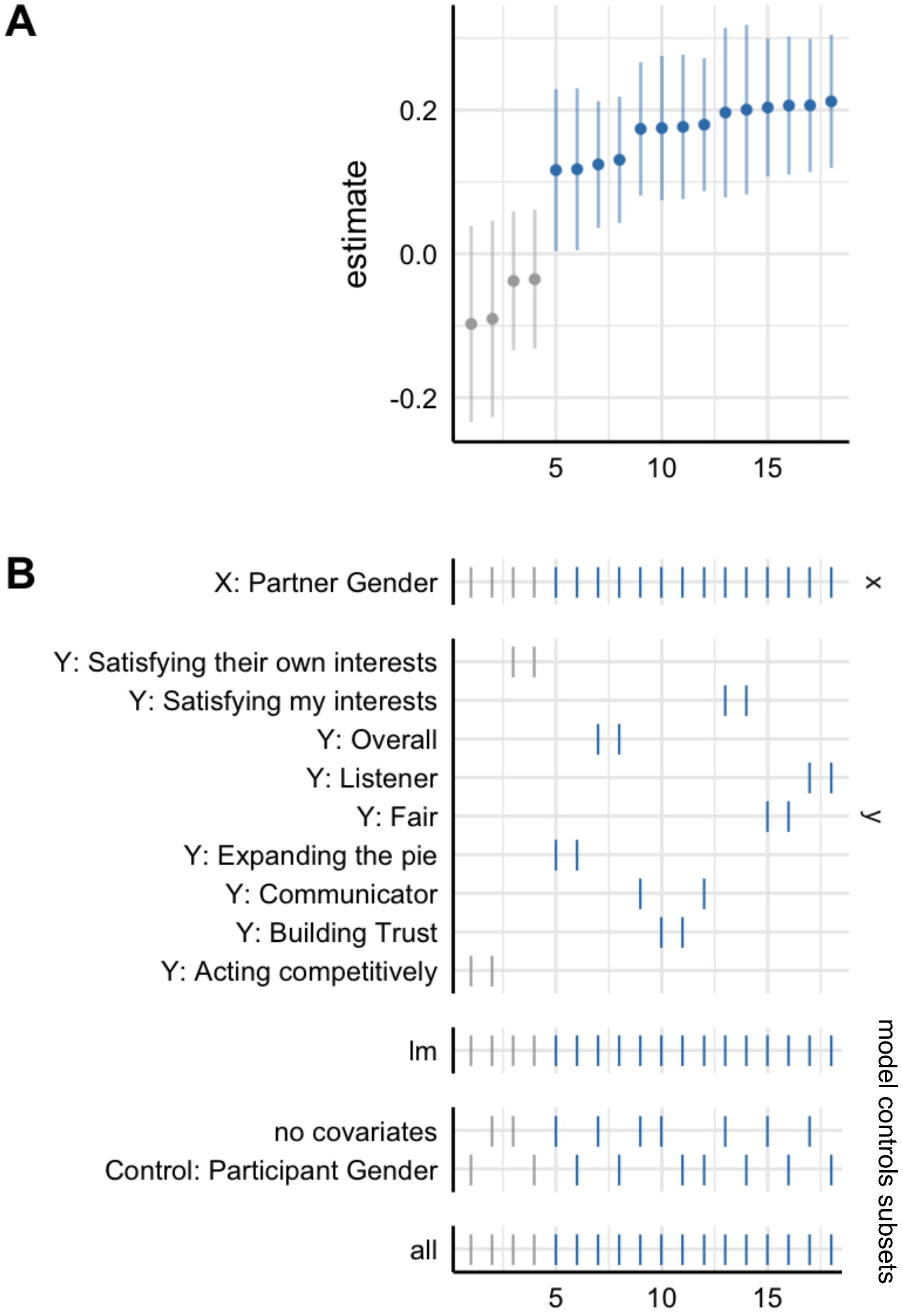

Figure S2. Specification curve analysis for multilevel models (Study 1).

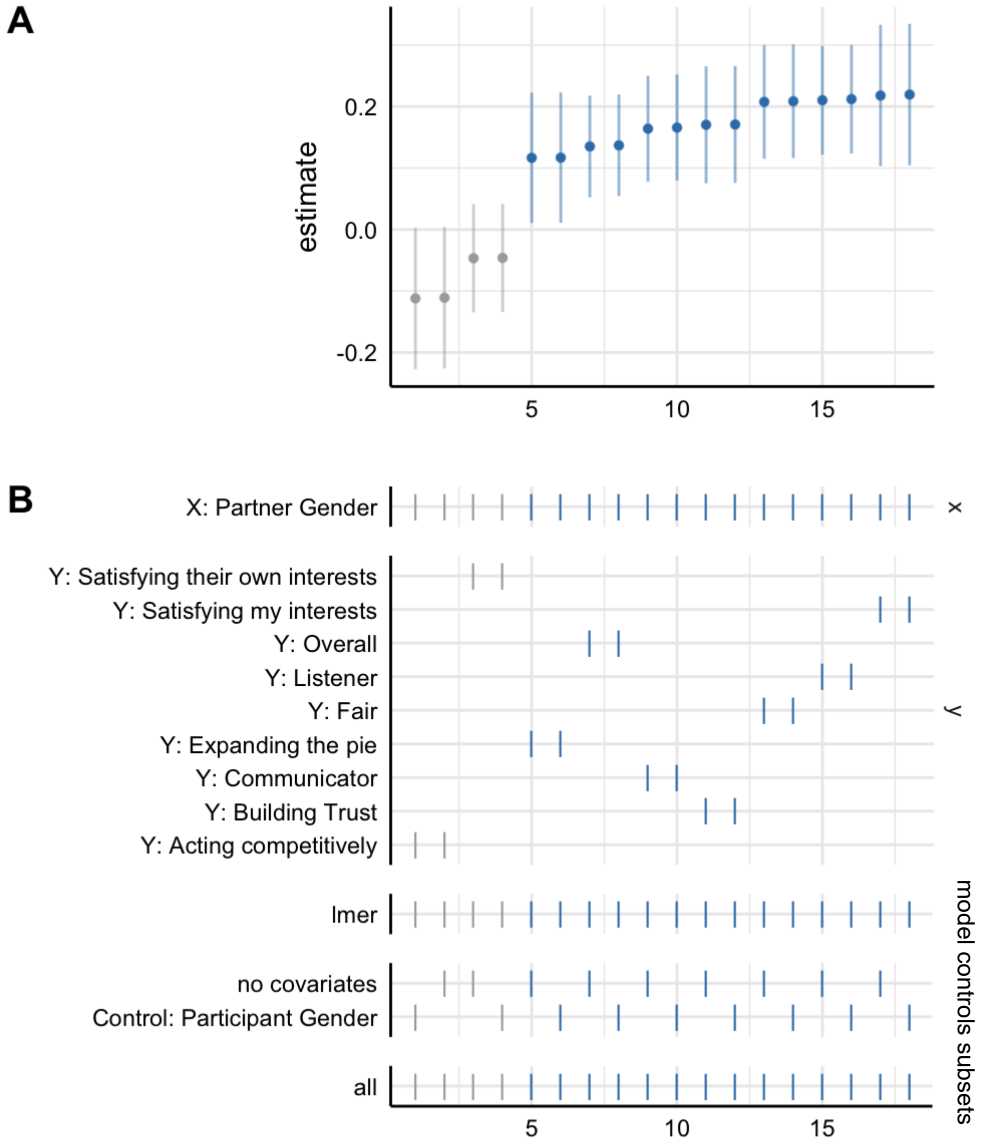

Figure S3. Example chat dialogue (Study 2 pretest).

Chat Messages

agent_2: Hello! I am excited for my trip, how about you?

agent_1: I am also very excited. Thank you for asking!

agent_2: I am in need of some water the most, what is your highest priority item?

agent_1: Water too

agent_2: OK, what is the item that is the next most important to you?

agent_1: Well, I need food for energy. So that is the next most important thing for me.

agent_2: OK, how about you take 2 water and 1 food and I firewood?

agent_1: Could I have 2 water and 2 food instead? There are 9 items. Obviously, one person will get 4 and the other 5. If I am getting 4, I would prefer 2 food items.

agent_2: I really will need 2 of either the water or the food, you can decide if you would like.

agent_1: Then can I get 2 water, 1 food, and 2 firewood?

agent_2: Sure, that sounds good!

agent_1: Okay, then we have a deal.

agent_2: Submit-Deal

agent_1: Accept-Deal

Figure S4. Serial mediation model (Study 5).

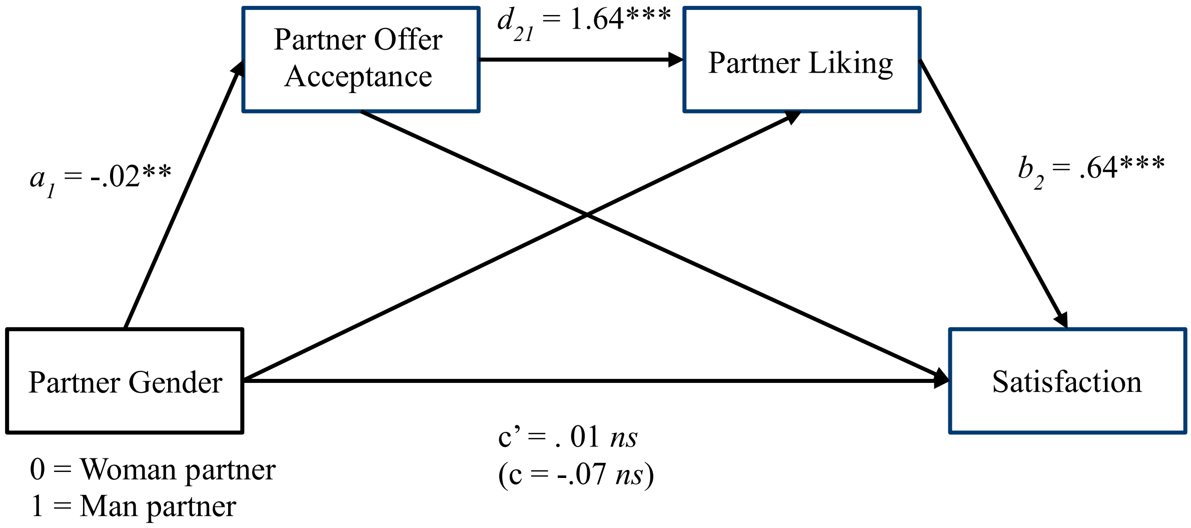

Note. This serial mediation model uses the offer acceptance measure, defined as the proportion of messages from their partner coded as "Offer Acceptance".

Figure S5. Simulation of women's advantage in repeat negotiations.

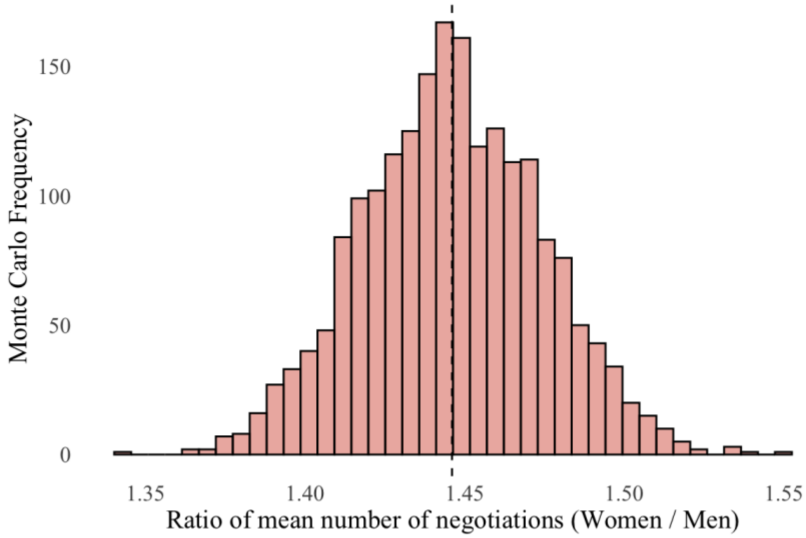

Note. The dotted line represents the mean ratio across simulations (M = 1.45). Therefore, on average, we'd expect women to have almost 45 percent more negotiations over time.

Table S1. Women partners are rated higher than men partners in building trust, fairness, and satisfying their partners' interests (Study 1).

	Partner: Building Trust			Partner: Fair			Partner: Satisfying my interests			Partner: Satisfying their own interests			Partner: Acting competitively		
Predictors	*Estimates*	*CI*	*p*	*Estimates*	*CI*	*p*	*Estimates*	*CI*	*p*	*Estimates*	*CI*	*p*	*Estimates*	*CI*	*p*
Participant Gender (ref = woman)	-0.05	-0.21 – 0.11	0.515	-0.08	-0.22 – 0.06	0.267	-0.11	-0.28 – 0.06	0.193	-0.08	-0.25 – 0.09	0.360	0.27	-0.01 – 0.55	0.063
Partner Gender (ref = woman)	-0.17	-0.27 – -0.08	**<0.001**	-0.21	-0.30 – -0.12	**<0.001**	-0.22	-0.33 – -0.10	**<0.001**	0.05	-0.04 – 0.13	0.305	0.11	-0.00 – 0.23	0.057
Constant	5.74	5.61 – 5.88	**<0.001**	5.88	5.75 – 6.00	**<0.001**	5.43	5.28 – 5.58	**<0.001**	5.70	5.55 – 5.84	**<0.001**	3.87	3.64 – 4.11	**<0.001**
Num. obs.	2,321			2,319			2,307			2,318			2,319		
Num. participants	231			231			231			231			231		
Marginal R^2 / Conditional R^2	0.005 / 0.168			0.009 / 0.137			0.007 / 0.110			0.002 / 0.239			0.007 / 0.356		

Note. We ran a multilevel linear model with a random intercept for the participant who completed the survey, regressing participant gender and partner gender on each relational outcome measure.

Table S2. Women partners are rated higher than men partners in expanding the pie, communicating, listening, and overall effectiveness (Study 1).

	Partner: Expanding the pie			Partner: Communicator			Partner: Listener			Partner: Overall		
Predictors	*Estimates*	*CI*	*p*	*Estimates*	*CI*	*p*	*Estimates*	*CI*	*p*	*Estimates*	*CI*	*p*
Participant Gender (ref = woman)	-0.03	-0.21 – 0.15	0.753	-0.19	-0.35 – -0.04	**0.014**	-0.17	-0.31 – -0.03	**0.021**	-0.20	-0.34 – -0.06	**0.005**
Partner Gender (ref = woman)	-0.12	-0.22 – -0.01	**0.031**	-0.17	-0.25 – -0.08	**<0.001**	-0.21	-0.30 – -0.12	**<0.001**	-0.14	-0.22 – -0.05	**0.001**
Constant	5.27	5.11 – 5.43	**<0.001**	6.02	5.89 – 6.15	**<0.001**	6.05	5.93 – 6.18	**<0.001**	5.92	5.80 – 6.05	**<0.001**
Num. obs.	2,317			2,318			2,317			2,318		
Num. participants	231			231			231			231		
Marginal R^2 / Conditional R^2	0.002 / 0.178			0.012 / 0.200			0.014 / 0.161			0.012 / 0.185		

Note. We ran a multilevel linear model with a random intercept for the participant who completed the survey, regressing participant gender and partner gender on each relational outcome measure.

Table S3. Participant Gender x Partner Gender interaction does not predict any subjective value measures (Study 1).

	Partner: Building Trust			Partner: Fair			Partner: Satisfying my interests			Partner: Satisfying their own interests			Partner: Acting competitively		
Predictors	*Estimates*	*CI*	*p*	*Estimates*	*CI*	*p*	*Estimates*	*CI*	*p*	*Estimates*	*CI*	*p*	*Estimates*	*CI*	*p*
Participant Gender (ref = woman)	-0.08	-0.28 – 0.13	0.459	-0.05	-0.23 – 0.14	0.628	-0.18	-0.40 – 0.05	0.121	-0.09	-0.29 – 0.12	0.406	0.17	-0.15 – 0.48	0.309
Partner Gender (ref = woman)	-0.19	-0.35 – -0.04	**0.015**	-0.17	-0.33 – -0.02	**0.025**	-0.29	-0.47 – -0.10	**0.003**	0.04	-0.11 – 0.18	0.594	0.01	-0.18 – 0.20	0.930
Participant Gender X Partner Gender	0.04	-0.16 – 0.23	0.713	-0.06	-0.25 – 0.14	0.566	0.11	-0.13 – 0.34	0.383	0.01	-0.17 – 0.19	0.906	0.16	-0.07 – 0.40	0.175
Constant	5.76	5.60 – 5.92	**<0.001**	5.86	5.71 – 6.00	**<0.001**	5.47	5.29 – 5.65	**<0.001**	5.70	5.54 – 5.87	**<0.001**	3.94	3.69 – 4.19	**<0.001**
Num. obs.	2,321			2,319			2,307			2,318			2,319		
Num. participants	231			231			231			231			231		
Marginal R^2 / Conditional R^2	0.005 / 0.168			0.009 / 0.137			0.007 / 0.110			0.002 / 0.239			0.008 / 0.358		

Note. We ran a multilevel linear model with a random intercept for the participant who completed the survey, regressing participant gender, partner gender, and their interaction on each relational outcome measure.

Table S4. Participant Gender x Partner Gender interaction does not predict any subjective value measures (Study 1).

	Partner: Expanding the pie			Partner: Communicator			Partner: Listener			Partner: Overall		
Predictors	*Estimates*	*CI*	*p*	*Estimates*	*CI*	*p*	*Estimates*	*CI*	*p*	*Estimates*	*CI*	*p*
Participant Gender (ref = woman)	-0.16	-0.39 – 0.07	0.169	-0.17	-0.36 – 0.02	0.072	-0.14	-0.33 – 0.04	0.122	-0.14	-0.32 – 0.04	0.121
Partner Gender (ref = woman)	-0.25	-0.42 – -0.08	**0.005**	-0.15	-0.29 – -0.01	**0.041**	-0.19	-0.33 – -0.04	**0.011**	-0.07	-0.21 – 0.06	0.282
Participant Gender X Partner Gender	0.21	-0.01 – 0.43	0.060	-0.03	-0.21 – 0.15	0.745	-0.04	-0.22 – 0.14	0.673	-0.10	-0.27 – 0.07	0.255
Constant	5.35	5.17 – 5.53	**<0.001**	6.01	5.86 – 6.16	**<0.001**	6.04	5.89 – 6.18	**<0.001**	5.88	5.74 – 6.02	**<0.001**
Num. obs.	2,317			2,318			2,317			2,318		
Num. participants	231			231			231			231		
Marginal R^2 / Conditional R^2	0.003 / 0.180			0.012 / 0.200			0.014 / 0.161			0.013 / 0.185		

Note. We ran a multilevel linear model with a random intercept for the participant who completed the survey, regressing participant gender, partner gender, and their interaction on each relational outcome measure.

Table S5. Participant gender and partner gender predicting self-evaluations (Study 1).

	Self: Building Trust			Self: Fair			Self: Satisfying my interests			Self: Satisfying their own interests			Self: Acting competitively		
Predictors	*Estimates*	*CI*	*p*	*Estimates*	*CI*	*p*	*Estimates*	*CI*	*p*	*Estimates*	*CI*	*p*	*Estimates*	*CI*	*p*
Participant Gender (ref = woman)	-0.08	-0.24 – 0.08	0.344	-0.11	-0.25 – 0.04	0.152	-0.13	-0.32 – 0.05	0.163	-0.03	-0.20 – 0.14	0.714	0.34	0.06 – 0.62	**0.018**
Partner Gender (ref = woman)	-0.06	-0.14 – 0.02	0.169	-0.03	-0.11 – 0.06	0.515	0.03	-0.07 – 0.13	0.556	-0.07	-0.16 – 0.01	0.100	-0.09	-0.19 – 0.02	0.104
Constant	5.40	5.26 – 5.54	**<0.001**	5.54	5.41 – 5.66	**<0.001**	5.09	4.93 – 5.25	**<0.001**	5.71	5.56 – 5.85	**<0.001**	4.13	3.90 – 4.37	**<0.001**
Num. obs.		2,280			2,278			2,277			2,278			2,276	
Num. participants		231			231			231			231			231	
Marginal R^2 / Conditional R^2		0.002 / 0.244			0.003 / 0.182			0.003 / 0.214			0.001 / 0.220			0.012 / 0.413	

Table S6. Participant gender and partner gender predicting self-evaluations (Study 1).

	Self: Expanding the pie			Self: Communicator			Self: Listener			Self: Overall		
Predictors	*Estimates*	*CI*	*p*	*Estimates*	*CI*	*p*	*Estimates*	*CI*	*p*	*Estimates*	*CI*	*p*
Participant Gender (ref = woman)	-0.00	-0.17 – 0.17	0.997	-0.16	-0.32 – 0.00	0.055	-0.22	-0.38 – -0.06	**0.007**	-0.14	-0.30 – 0.01	0.068
Partner Gender (ref = woman)	-0.04	-0.13 – 0.05	0.419	-0.04	-0.12 – 0.04	0.303	-0.04	-0.12 – 0.04	0.335	-0.09	-0.17 – -0.01	**0.036**
Constant	5.34	5.19 – 5.48	**<0.001**	5.54	5.40 – 5.68	**<0.001**	5.72	5.58 – 5.85	**<0.001**	5.44	5.31 – 5.57	**<0.001**
Num. obs.	2,277			2,278			2,279			2,278		
Num. participants	231			231			231			231		
Marginal R^2 / Conditional R^2	0.000 / 0.212			0.006 / 0.279			0.011 / 0.276			0.006 / 0.224		

Table S7. Participant Gender x Partner Gender interaction predicting self-evaluations (Study 1).

	Self: Building Trust			Self: Fair			Self: Satisfying my interests			Self: Satisfying their own interests			Self: Acting competitively		
Predictors	*Estimates*	*CI*	*p*	*Estimates*	*CI*	*p*	*Estimates*	*CI*	*p*	*Estimates*	*CI*	*p*	*Estimates*	*CI*	*p*
Participant Gender (ref = woman)	-0.00	- 0.19 – 0.19	0.972	-0.06	- 0.24 – 0.13	0.551	-0.02	- 0.25 – 0.20	0.849	-0.11	-0.31 – 0.09	0.286	0.23	- 0.09 – 0.54	0.156
Partner Gender (ref = woman)	0.02	- 0.12 – 0.15	0.791	0.02	- 0.11 – 0.16	0.734	0.14	- 0.02 – 0.31	0.090	-0.16	-0.30 – - 0.01	**0.038**	-0.20	-0.38 – - 0.03	**0.022**
Participant Gender X Partner Gender	-0.12	- 0.29 – 0.05	0.164	-0.08	- 0.26 – 0.09	0.353	-0.18	- 0.39 – 0.03	0.092	0.13	-0.06 – 0.31	0.175	0.18	- 0.04 – 0.40	0.102
Constant	5.35	5.20 – 5.51	**<0.001**	5.50	5.36 – 5.65	**<0.001**	5.02	4.84 – 5.20	**<0.001**	5.76	5.60 – 5.92	**<0.001**	4.21	3.96 – 4.46	**<0.001**
Num. obs.		2,280			2,278			2,277			2,278			2,276	
Num. participants		231			231			231			231			231	
Marginal R^2 / Conditional R^2		0.003 / 0.244			0.003 / 0.182			0.004 / 0.215			0.002 / 0.221			0.013 / 0.414	

Table S8. Participant Gender x Partner Gender interaction predicting self-evaluations (Study 1).

	Self: Expanding the pie			Self: Communicator			Self: Listener			Self: Overall		
Predictors	*Estimates*	*CI*	*p*	*Estimates*	*CI*	*p*	*Estimates*	*CI*	*p*	*Estimates*	*CI*	*p*
Participant Gender (ref = woman)	-0.00	-0.21 – 0.20	0.963	-0.16	-0.35 – 0.03	0.099	-0.24	-0.42 – -0.05	**0.015**	-0.15	-0.34 – 0.04	0.111
Partner Gender (ref = woman)	-0.04	-0.19 – 0.11	0.580	-0.04	-0.17 – 0.09	0.516	-0.05	-0.18 – 0.07	0.400	-0.10	-0.23 – 0.04	0.162
Participant Gender X Partner Gender	0.01	-0.18 – 0.20	0.939	0.00	-0.16 – 0.16	0.978	0.03	-0.13 – 0.19	0.749	0.01	-0.16 – 0.18	0.877
Constant	5.34	5.17 – 5.50	**<0.001**	5.54	5.39 – 5.69	**<0.001**	5.73	5.58 – 5.87	**<0.001**	5.45	5.30 – 5.59	**<0.001**
Num. obs.		2,277			2,278			2,279			2,278	
Num. participants		231			231			231			231	
Marginal R^2 / Conditional R^2		0.000 / 0.212			0.006 / 0.279			0.011 / 0.276			0.006 / 0.224	

Description of Survey Materials

For Study 1, students completed survey responses evaluating their partner and then themselves for each survey. Some survey questions have redacted text to avoid indicating the university where the data was collected. Note: Trustworthy items were only included at the end of the second semester of data, which is why this item is not included in the analyses and survey file. Participants also had the option to write open-ended feedback to partners. The complete survey (.qsf file) is available on the OSF page: https://osf.io/52qwc/?view_only=a10eade50990460ba7b0c5e0b7401ef3

For Study 2 pretest, due to the length of the survey, we have included an example. Participants were randomly assigned to view five transcripts. The complete survey (.qsf file) is available on the OSF page: https://osf.io/52qwc/?view_only=a10eade50990460ba7b0c5e0b7401ef3

For Study 3, due to the length of the surveys, we have included an example screenshot. Participants were randomly assigned to view five transcripts. The complete surveys (each condition is a separate survey) (.qsf files) are available on the OSF page: https://osf.io/52qwc/?view_only=a10eade50990460ba7b0c5e0b7401ef3

For Study 4, due to the length of the survey, we have included an example screenshot. Participants were randomly assigned to view five transcripts. The complete survey (.qsf file) is available on the OSF page: https://osf.io/52qwc/?view_only=a10eade50990460ba7b0c5e0b7401ef3

Study 1

NEGOTIATION FEEDBACK SURVEY

Please select the negotiation that you just completed.

Please enter your (@REDACTED.edu) REDACTED email

Please select your OWN name from the class roster list below. (If your name is not on the list, scroll to the bottom and click "My name is not on the list").

(Note: Names are organized by section and alphabetically by last name)

>>

Please select your NEGOTIATION PARTNER'S name from the class roster list below. (If their name is not on the list, scroll to the bottom and click "My partner's name is not on the list").

If you negotiated with more than one person, just select one person toward whom to direct this feedback. (You may fill out multiple surveys).

<<   >>

Please rate your negotiation partner on the following statements. Higher scores indicate greater agreement with this statement; lower scores indicate disagreement with the statement.

If any of these statements are not applicable to the negotiation, you may leave them blank.

	1 Not at all	2	3	4 Somewhat	5	6	7 Very Much
My negotiation partner was effective in building trust.	○	○	○	○	○	○	○
My negotiation partner was fair.	○	○	○	○	○	○	○
My negotiation partner seemed concerned with satisfying my interests.	○	○	○	○	○	○	○
My negotiation partner looked after his or her own interests.	○	○	○	○	○	○	○
My negotiation partner acted very competitively.	○	○	○	○	○	○	○
My negotiation partner tried to create value and expand the pie.	○	○	○	○	○	○	○
My negotiation partner was an active, effective **communicator** during the negotiation.	○	○	○	○	○	○	○
My negotiation partner was an active, effective **listener** during the negotiation.	○	○	○	○	○	○	○
My negotiation partner was effective overall.	○	○	○	○	○	○	○

Please rate YOURSELF on the following statements. Higher scores indicate greater agreement with this statement; lower scores indicate disagreement with the statement.

If these statements are not applicable to the negotiation, you may leave them blank.

	1 Not at all	2	3	4 Somewhat	5	6	7 Very Much
I was effective in building trust.	○	○	○	○	○	○	○
I was fair.	○	○	○	○	○	○	○
I was concerned with satisfying my **negotiation partner's** interests.	○	○	○	○	○	○	○
I looked after my own interests.	○	○	○	○	○	○	○
I acted very competitively.	○	○	○	○	○	○	○
I tried to create value and expand the pie.	○	○	○	○	○	○	○
I was an active, effective **communicator** during the negotiation.	○	○	○	○	○	○	○
I was an active, effective **listener** during the negotiation.	○	○	○	○	○	○	○
I was effective overall.	○	○	○	○	○	○	○

Given the way your partner negotiated, would you want to work with your partner again?

○ Yes

○ No

Please provide feedback on what your partner did well and not as well. Focus on behavior and provide an example, if appropriate.

Note the following areas to reference:

- Anchoring (appropriate--edge of reasonableness, timely, wiped away, extreme)
- Packaging (included appropriate issues, address interests, balanced)
- Concessions (amount, timing, principled, multiple)
- Asking enough questions/showed interest in other needs
- Revealing enough information; timing of information shared
- Shared interests; made other comfortable revealing interests
- Used reciprocity in revealing information and making concessions
- Behaviors that showed person was listening and understanding; acknowledging other's points
- Non-verbal: tone of voice, eye contact, gestures, body movements
- Appropriate expressions/reading of non-verbals
- Face saving
- Sharing/understanding priorities
- Paraphrasing/summarizing where appropriate
- Building trust/repairing trust
- Managing emotions of self/other
- Effectiveness of influence tactics
- Appeared flexible/too rigid
- Disclosed appropriate information
- Appeared honest/trustworthy--explain why
- Managing different negotiation styles well (accommodator, competitor, etc.)
- Used bridging, logrolling, pie-expansion or cost cutting techniques
- Facilitated the conversation in a group/stepped in to guide or move the group along
- Building upon ideas of others in a team

Please provide feedback on the things your partner did well. Please be thoughtful and thorough when describing what your partner did well.

Please provide feedback on the things your partner could do better. This should be constructive criticism. Please be thoughtful and thorough when describing your partner's potential improvements.

Study 2 pretest

On the pages that follow, you will be asked to read and evaluate chat conversations from an online negotiation in which participants were negotiating campsite resources. These are *real* conversation so they may include typos and misspellings.

Participants were encouraged to use emojis and had the option to use the following emojis in conversation:

sad face :(
angry face >:(
surprised face :O
smiling face :)

→

Each agent self-identified as a man or woman. Please do your best to guess the gender of each agent and explain why. There are no right or wrong answers, we just ask that you answer honestly.

→

Chat Messages

agent_1: Hi, how are you, I'm really excited about my trip, how about you?

agent_2: I am excited as well :)! I can't wait to finally get some fresh air!

agent_1: Me too! Speaking of which, I'd really like to be able to have as long of a fire as possible, sing songs and stuff. I'd like all the extra firewood, I'd be okay with only one extra food and water.

agent_2: Oh, ironically I was going to ask the same. The site I camp at is very cold at night, not to mention all the food I brought requires cooking :(. Maybe since we both are in need of wood we can keep it, but I may require an extra water. I'd be willing to lend an extra ration of food for it however.

agent_1: So I would get 3 firewood, no water and 2 food?

agent_2: My bad, I misspoke. I'm still getting over my cold so please forgive me. I am proposing this-I would get 2 firewood, 1 water and 1 food. You would get 1 firewood, 2 water and 2 food. How does that sound?

agent_1: Sure, I am good with that deal.

agent_2: I am happy about that! I hope your trip goes great and you get much needed relaxation!

agent_1: same to you

agent_2: I am sure I will have a great camping trip!

agent_1: Submit-Deal

agent_2: Accept-Deal

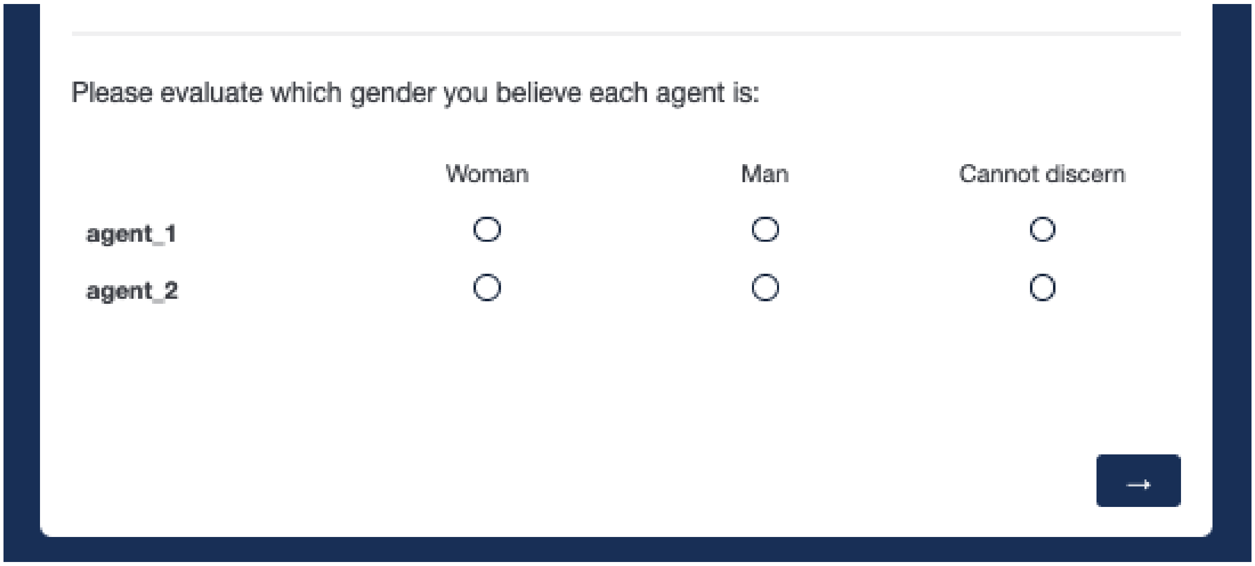
Please evaluate which gender you believe each agent is:

	Woman	Man	Cannot discern
**agent_1**	○	○	○
**agent_2**	○	○	○

→

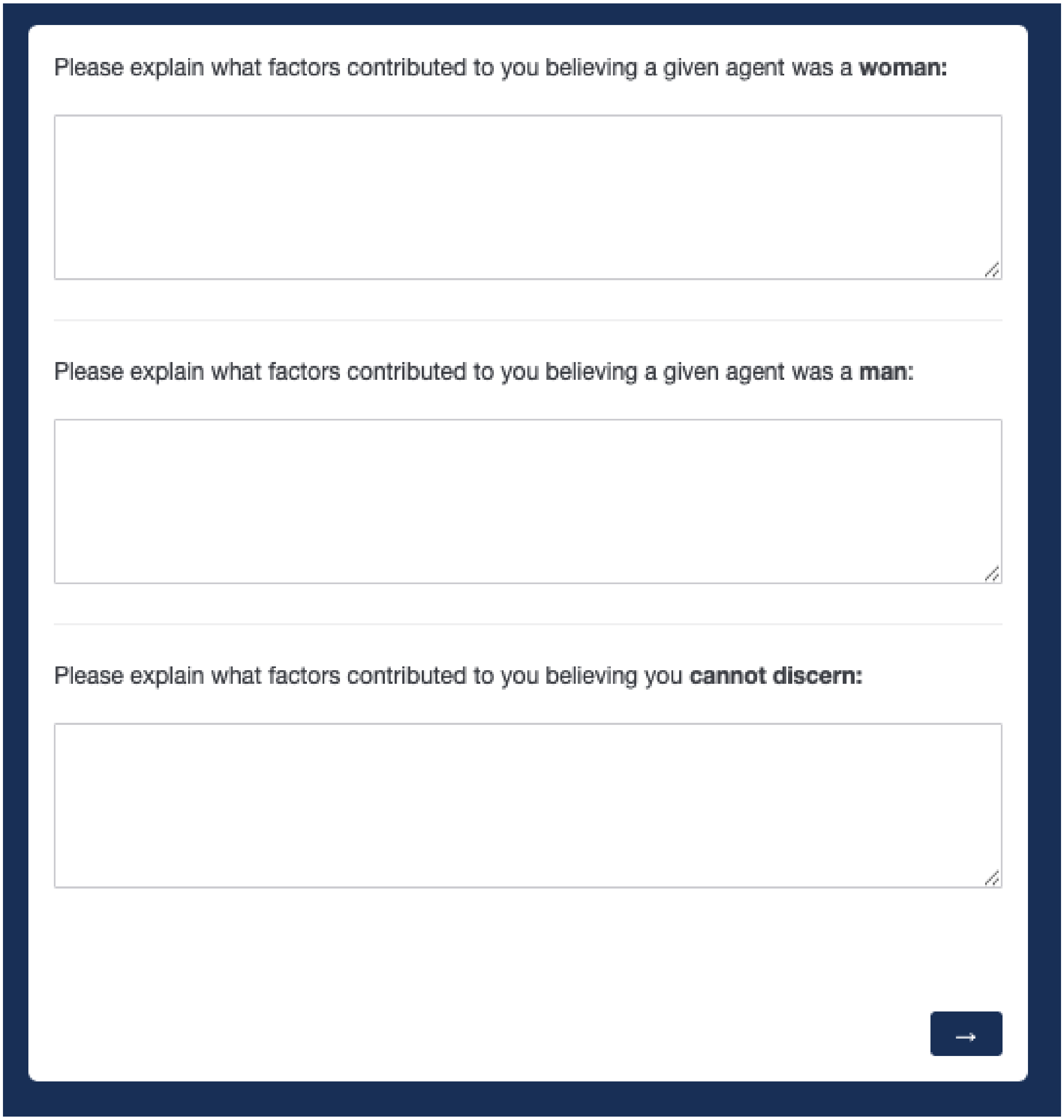
Please explain what factors contributed to you believing a given agent was a **woman:**

Please explain what factors contributed to you believing a given agent was a **man**:

Please explain what factors contributed to you believing you **cannot discern:**

→

After each conversation, partners rated how much they liked their negotiation partner. The rating options were:

Extremely dislike, Slightly dislike, Undecided, Slightly like, Extremely like.

For the next series of chat conversations, you will be providing your own explanations as to why participants rated their partner as such in 'liking'.

→

Chat Messages

agent_1: Hello there. I am excited about this camping trip!

agent_2: I'm also excited, I love the outdoors!

agent_1: My favorite thing to do is sing songs and tell stories around a campfire. Consequently, I was really hoping to get some additional packages of firewood. :)

agent_2: I do love sing alongs around the campfire. I usually go hiking and it requires a lot of energy and fuel. Maybe I can get additional food packages?

agent_1: You'll definitely need some additional calories if you want to do extra hiking. I will give you all the additional food if you give me all the additional firewood.

agent_2: Sounds like a deal, maybe split the water packages evenly. Can't be dehyradated while camping :)

agent_1: Agreed. Your fun trip could turn into something quite dangerous without that hydration.

agent_2: Of course, that won't be fun. :(So we have agreed

agent_1: Mostly - since we cannot split the water 1-1, do you mind if I take the extra one? :)

agent_2: That's fine, you might need it more.

agent_1: Submit-Deal

agent_2: Accept-Deal

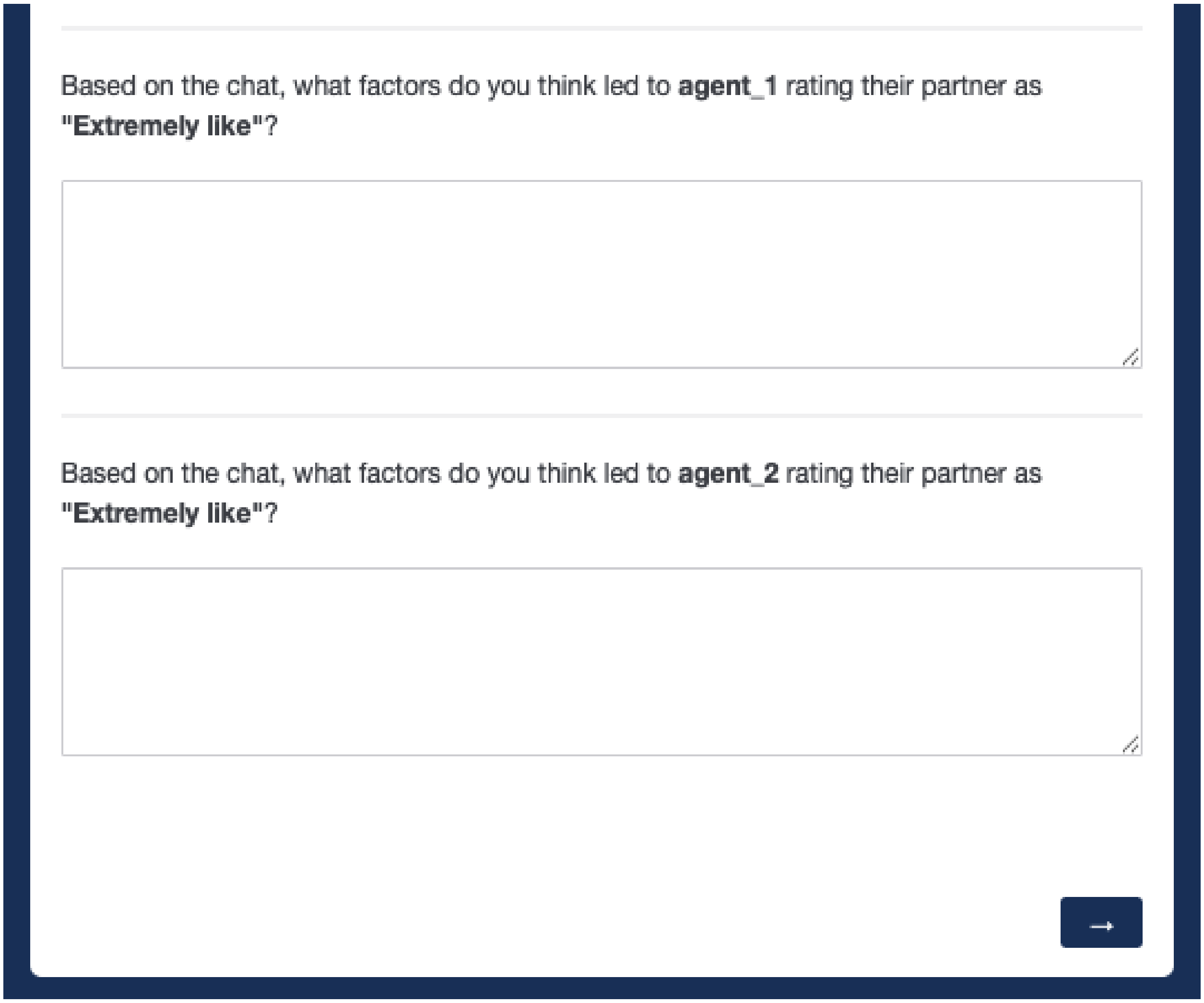
Based on the chat, what factors do you think led to **agent_1** rating their partner as **"Extremely like"**?

Based on the chat, what factors do you think led to **agent_2** rating their partner as **"Extremely like"**?

→

Please answer a few questions about yourself:

Please indicate your age (in years).

Gender

- ◯ Man
- ◯ Woman
- ◯ Nonbinary/Genderfluid
- ◯ Something not listed
- ◯ Prefer not to answer

Which of the following racial/ethnic group(s) do you consider yourself a member of? You may check multiple groups.

- ☐ White/European American
- ☐ Black/African American
- ☐ East Asian/East Asian American
- ☐ Southeast Asian/Southeast Asian American
- ☐ South Asian/South Asian American
- ☐ Latino/Hispanic American
- ☐ Native/American Indian
- ☐ Middle Eastern/Arab American
- ☐ Something not listed

What is the highest level of school you have completed or the highest degree you have received?

- ◯ Less than high school degree
- ◯ High school graduate (high school diploma or equivalent including GED)
- ◯ Some college but no degree
- ◯ Associate degree in college (2-year)
- ◯ Bachelor's degree in college (4-year)
- ◯ Master's degree
- ◯ Doctoral degree
- ◯ Professional degree (JD, MD)

How politically conservative or liberal do you think you are?

Very Conservative -4	-3	-2	-1	1	2	3	Very Liberal 4
◯	◯	◯	◯	◯	◯	◯	◯

Is English your native language?

- ◯ Yes
- ◯ No

→

What do you think this study was about?

→

Do you have any comments about this study? If so, please write them below.

→

We appreciate the time you took on this survey!
So we have the best information possible, please let us know if you read each chat carefully.

Please be honest. You will receive credit REGARDLESS of your answers.

- ○ Yes, I read the entire chats carefully
- ○ No, I DID NOT read the entire chats carefully

→

Study 3 – Control Condition

On the pages that follow, you will be asked to read and evaluate chat transcripts from an online negotiation in which participants were negotiating campsite resources. These are real conversations so they may include typos and misspellings.

Participants were encouraged to use emojis and had the option to use the following emojis in conversation:

sad face :(
angry face >:(
surprised face :O
smiling face :)

Survey Completion
0% 100%

→

Each agent self-identified as a man or woman. Please do your best to imagine you are participating in the following negotiations. There are no right or wrong answers, we just ask that you answer honestly.

Survey Completion
0% 100%

→

You are going to read **5 transcripts**. Each transcript will appear on one page, followed by questions assessing your impressions of one of the negotiators on the next page. You will not be able to return to the transcript once you advance the page, so please read carefully.

Survey Completion
0% 100%

→

Please read the following conversation and imagine that **YOU are agent_2**. agent_1 is your negotiation partner:

agent_1
: Hi! How are you doing?

agent_2
: I'm well! Excited for this camping trip.

agent_1
: Same! I'm a little worried about some of my supplies though. What about you?

agent_2
: Likewise! What are you most in need of?

agent_1
: Well, I'm camping up on a snowy mountain and I gave some of my food away to hikers who were passing me and were very tired and hungry, so I'd really like to get some more food. Firewood would be useful too, although I do have a thick coat and a sleeping bag. I plan on melting the snow to get water, so I don't think I'll need that too much. And you?

agent_2
: I have the same needs. I have a low metabolism, so I need food and firewood to keep me warm and energized. Water I plan on filtering elsewhere.

agent_1
: Ok, well, I'd like to help. How about if I take 2 food and 1 firewood and 2 water, and you can keep the rest?

agent_2
: Would you mind if you got 3 food and 1 water and I got 3 firewood and 2 water?

agent_1
: I'd like some firewood though... Would 3 food and 1 firewood for me work?

agent_2
: I would probably do that, yes

agent_1
: Submit-Deal

agent_2
: Accept-Deal

In your opinion, how...

	Not at all 1	2	3	4	5	6	Extremely 7
efficient is agent_1?	○	○	○	○	○	○	○
confident is agent_1?	○	○	○	○	○	○	○
good-natured is agent_1?	○	○	○	○	○	○	○
warm is agent_1?	○	○	○	○	○	○	○
	Not at all 1	2	3	4	5	6	Extremely 7
skillful is agent_1?	○	○	○	○	○	○	○
competent is agent_1?	○	○	○	○	○	○	○
friendly is agent_1	○	○	○	○	○	○	○
intelligent is agent_1?	○	○	○	○	○	○	○
	Not at all 1	2	3	4	5	6	Extremely 7
trustworthy is agent_1?	○	○	○	○	○	○	○
well-intentioned is agent_1?	○	○	○	○	○	○	○
sincere is agent_1?	○	○	○	○	○	○	○
capable is agent_1?	○	○	○	○	○	○	○

How much would you like agent_1?

Extremely dislike	Dislike a moderate amount	Dislike a little	Neither like nor dislike	Like a little	Like a moderate amount	Extremely like
○	○	○	○	○	○	○

How satisfied would you be with the negotiation outcome?

Extremely dissatisfied	Dissatisfied a moderate amount	Dissatisfied a little	Neither satisfied nor dissatisfied	Satisfied a little	Satisfied a moderate amount	Extremely satisfied
○	○	○	○	○	○	○

How much would you like to negotiate with agent_1 in the future?

Extremely dislike	Dislike a moderate amount	Dislike a little	Neither like nor dislike	Like a little	Like a moderate amount	Extremely like
○	○	○	○	○	○	○

Please answer a few questions about yourself:

Please indicate your age (in years).

Gender

- ○ Man
- ○ Woman
- ○ Nonbinary/Genderfluid
- ○ Something not listed
- ○ Prefer not to answer

Which of the following racial/ethnic group(s) do you consider yourself a member of? You may check multiple groups.

- ☐ White/European American
- ☐ Black/African American
- ☐ East Asian/East Asian American
- ☐ Southeast Asian/Southeast Asian American
- ☐ South Asian/South Asian American
- ☐ Latino/Hispanic American
- ☐ Native/American Indian
- ☐ Middle Eastern/Arab American
- ☐ Something not listed

What is the highest level of school you have completed or the highest degree you have received?

- ◯ Less than high school degree
- ◯ High school graduate (high school diploma or equivalent including GED)
- ◯ Some college but no degree
- ◯ Associate degree in college (2-year)
- ◯ Bachelor's degree in college (4-year)
- ◯ Master's degree
- ◯ Doctoral degree
- ◯ Professional degree (JD, MD)

How politically conservative or liberal do you think you are?

Very Conservative -4	-3	-2	-1	1	2	3	Very Liberal 4
◯	◯	◯	◯	◯	◯	◯	◯

Is English your native language?

- ◯ Yes
- ◯ No

Survey Completion

0% 100%

→

What do you think this study was about?

Survey Completion
0% 100%

→

Do you have any comments about this study? If so, please write them below.

Survey Completion
0% 100%

→

We appreciate the time you took on this survey!
So we have the best information possible, please let us know if you read each chat carefully.

Please be honest. You will receive credit REGARDLESS of your answers.

- ◯ Yes, I read the entire chats carefully
- ◯ I read some but not all chats carefully
- ◯ No, I DID NOT read the entire chats carefully

Survey Completion
0% 100%

→

Study 3 – Gender Information Condition

[Instructions are the same as the Control Condition]

Please read the following conversation and imagine that **YOU are agent_1**. agent_2 is a woman:

agent_2
: Hello! I am excited for my trip, how about you?

agent_1
: I am also very excited. Thank you for asking!

agent_2
: I am in need of some water the most, what is your highest priority item?

agent_1
: Water too

agent_2
: OK, what is the item that is the next most important to you?

agent_1
: Well, I need food for energy. So that is the next most important thing for me.

agent_2
: OK, how about you take 2 water and 1 food and I firewood?

agent_1
: Could I have 2 water and 2 food instead? There are 9 items. Obviously, one person will get 4 and the other 5. If I am getting 4, I would prefer 2 food items.

agent_2
: I really will need 2 of either the water or the food, you can decide if you would like.

agent_1
: Then can I get 2 water, 1 food, and 2 firewood?

agent_2
: Sure, that sounds good!

agent_1
: Okay, then we have a deal.

agent_2
: Submit-Deal

agent_1
: Accept-Deal

[Measures are the same as Control Condition]

Study 4

On the pages that follow, you will be asked to read and evaluate chat transcripts from an online negotiation in which participants were negotiating campsite resources. These are real conversations so they may include typos and misspellings.

Participants were encouraged to use emojis and had the option to use the following emojis in conversation:

sad face :(
angry face >:(
surprised face :O
smiling face :)

Each agent self-identified as a man or woman. Please do your best to imagine you are participating in the following negotiations. There are no right or wrong answers, we just ask that you answer honestly.

You are going to read **5 transcripts**. Each transcript will appear on one page, followed by questions assessing your impressions of one of the negotiators on the next page. You will not be able to return to the transcript once you advance the page, so please read carefully.

Please read the following conversation and imagine that **YOU are agent_2**. agent_1 is your negotiation partner:

agent_1
: Hello. I really need the extra water so that I can remain safe while camping. I get dehydrated.

agent_2
: I'm traveling with kids and it's going to be really hot during day time. Want to make sure that everyone is safe. How about you let me keep two waters and all of the extra food. You can have all of the firewood.

agent_1
: I understand that you have kids, but I require extra water to avoid dehydrating and cramping. How about I keep 2 waters, 2 firewoods and one food.

agent_2
: How many people are you traveling with? Do you really need this much water? Let me keep two waters.

agent_1
: Yes. I need a lot of water to stay hydrated. I am traveling with two other people.

agent_2
: If you are keeping two waters can I get two of firewood and two of food?

agent_1
: You can definitely have two food. Will you be cooking a lot? I cook a lot which is why I wanted two of the firewood. :)

agent_2
: Yes, as I said I'm traveling with family so we will be doing a lot of cooking and sitting at the fire pit in the evening. That's why I need two firewood

agent_1
: Ok. I understand. I will let you have 2 firewood and 2 food. I will take the two waters, 1 firewood and 1 food.

agent_2
: Sounds great!

agent_1
: You have a deal. Thanks.

agent_2
: Submit-Deal

agent_1
: Accept-Deal

Please respond to each of the following questions.

	Strongly disagree	Disagree	Somewhat disagree	Neither agree nor disagree	Somewhat agree	Agree	Strongly agree
The negotiation process was fair.	○	○	○	○	○	○	○
I am satisfied with the ease (or difficulty) of reaching an agreement.	○	○	○	○	○	○	○
My counterpart considered my wishes, opinions, and needs.	○	○	○	○	○	○	○
The negotiation made me trust my counterpart.	○	○	○	○	○	○	○
I am satisfied with my own outcome in this negotiation.	○	○	○	○	○	○	○

	Strongly disagree	Disagree	Somewhat disagree	Neither agree nor disagree	Somewhat agree	Agree	Strongly agree
This negotiation positively impacted my self-image or impression of myself.	○	○	○	○	○	○	○
I feel like I forfeited or "lost" in this negotiation.	○	○	○	○	○	○	○
I behaved according to my own principles and values.	○	○	○	○	○	○	○
Overall, my counterpart made a positive impression on me.	○	○	○	○	○	○	○
The negotiation built a good foundation for a future relationship with my counterpart.	○	○	○	○	○	○	○

	Strongly disagree	Disagree	Somewhat disagree	Neither agree nor disagree	Somewhat agree	Agree	Strongly agree
I think the terms of my agreement were fair.	○	○	○	○	○	○	○
This negotiation made me feel more competent as a negotiator.	○	○	○	○	○	○	○
I "lost face" (i.e., damaged my sense of pride) in the negotiation.	○	○	○	○	○	○	○

How much would you like agent_1 to be your teammate in a future negotiation?

Extremely dislike	Dislike a moderate amount	Dislike a little	Neither like nor dislike	Like a little	Like a moderate amount	Extremely like
○	○	○	○	○	○	○

How much would you like agent_1 to be your negotiating counterpart in a win-lose, competitive negotiation?

Extremely dislike	Dislike a moderate amount	Dislike a little	Neither like nor dislike	Like a little	Like a moderate amount	Extremely like
○	○	○	○	○	○	○

How much would you like agent_1 to be your negotiating counterpart in a win-win, cooperative negotiation?

Extremely dislike	Dislike a moderate amount	Dislike a little	Neither like nor dislike	Like a little	Like a moderate amount	Extremely like
○	○	○	○	○	○	○

Recent research on decision making shows that choices are affected by context. Specifically, we are interested in whether you actually take the time to read the directions; if not, some results may not tell us very much about decision making in the real world. To show that you have read the instructions, please ignore the job attributes below and instead select "neither important nor unimportant" option as your answer for all attributes.

Please rate the extent to which each job attribute is important to you.

	Not at all important	Very unimportant	Somewhat unimportant	Neither important nor unimportant	Somewhat important	Very important	Extreme importa
Educational opportunities	○	○	○	○	○	○	○
Diverse colleagues	○	○	○	○	○	○	○
Challenging tasks	○	○	○	○	○	○	○

→

Please answer a few questions about yourself:

Please indicate your age (in years).

[ ]

Gender

- ◯ Man
- ◯ Woman
- ◯ Nonbinary/Genderfluid
- ◯ Something not listed

[ ]

- ◯ Prefer not to answer

Which of the following racial/ethnic group(s) do you consider yourself a member of? You may check multiple groups.

- ☐ White/European American
- ☐ Black/African American
- ☐ East Asian/East Asian American
- ☐ Southeast Asian/Southeast Asian American
- ☐ South Asian/South Asian American
- ☐ Latino/Hispanic American
- ☐ Native/American Indian
- ☐ Middle Eastern/Arab American
- ☐ Something not listed

What is the highest level of school you have completed or the highest degree you have received?

- ◯ Less than high school degree
- ◯ High school graduate (high school diploma or equivalent including GED)
- ◯ Some college but no degree
- ◯ Associate degree in college (2-year)
- ◯ Bachelor's degree in college (4-year)
- ◯ Master's degree
- ◯ Doctoral degree
- ◯ Professional degree (JD, MD)

How politically conservative or liberal do you think you are?

Very Conservative -4	-3	-2	-1	1	2	3	Very Liberal 4
◯	◯	◯	◯	◯	◯	◯	◯

Is English your native language?

- ◯ Yes
- ◯ No

What do you think this study was about?

[   ]

→

Do you have any comments about this study? If so, please write them below.

→

We appreciate the time you took on this survey!
So we have the best information possible, please let us know if you read each chat carefully.

Please be honest. You will receive credit REGARDLESS of your answers.

- ◯ Yes, I read the entire chats carefully
- ◯ I read some but not all chats carefully
- ◯ No, I DID NOT read the entire chats carefully
